# Medicinal plants traded in Hakka communities of southeastern Guangxi, China

**DOI:** 10.1186/s13002-025-00796-y

**Published:** 2025-07-11

**Authors:** Yongqing Liufu, Yaozhang Xie, Min Shao, Qiongyao Fu, Zhongxin Duan, Dipak Khadka, Binsheng Luo

**Affiliations:** 1 Natural History Museum of Guangxi, Nanning, 530012 China; 2 Lushan Botanical Garden, Jiangxi Province and Chinese Academy of Sciences, Lushan, 332900 China; 3 Guangdong Key Laboratory for Innovative Development and Utilization of Forest Plant Germplasm, College of Forestry and Landscape Architecture, South China Agricultural University, Guangzhou, 510642 China

**Keywords:** Hakka, Medicinal plants, Traditional markets, Ethnobotany

## Abstract

**Background:**

The Hakka are one of the major subgroups of the Han  Chinese in China, with a unique migration history and a rich traditional medicine system. Traditional markets serve as centers for the exchange of Hakka medicinal culture and play a vital role in maintaining residents’ health. However, medicinal plants traded in traditional Hakka markets have not been documented in southeastern Guangxi, China.

**Methods:**

We documented the medicinal plants traded in the market of Hakka communities in southeastern Guangxi, China, by interviewing 106 traditional shopkeepers. Relative frequency of citation (RFC) and relative importance value (RI) were used to evaluate the most medicinal plants traded in the market of Hakka communities, and the Jaccard index of similarity (JI) was used to assess the similarity of plants used by Hakka from different communities in China.

**Results:**

We documented 305 medicinal plant species, with the Fabaceae family being the most represented (27 species). In terms of life form, herbs constituted the largest group, accounting for 130 species (42.62%). Regarding plant parts used, leaves were the most commonly utilized, reported for 122 species (32.36%). The recorded medicinal plants exhibit 63 therapeutic effects and are used to treat 117 ailments across 14 disease categories. The most frequently cited medicinal use was for the treatment of physical trauma (126 species, 41.31%), followed by digestive disorders (96 species, 31.47%), skin diseases (90 species, 29.51%), and detoxification (79 species, 25.90%). Twenty-eight plant species had a relative citation frequency (RFC) greater than 0.5, and 23 species had a relative importance (RI) value of ≥ 1.25. A comparative analysis with medicinal plants recorded in Hakka communities of Guangdong and Ganzhou revealed some similarities, with Jaccard index (JI) values of 0.10 and 0.06, respectively.

**Conclusion:**

The local shopkeepers of traditional markets in Hakka communities in southeastern Guangxi are prosperous in their knowledge of medicinal plant use. We recommend conserving these medicinal plants of local people by promoting the traditional market, which fosters people's livelihood through income generation and supports the sustainability of the traditional knowledge system.

## Introduction

The Hakka people, a subgroup of the Han nationality, are believed to have originated in the Central Plains of China [1]. Beginning around 300 A.D., they gradually migrated southward in response to wars and natural disasters, eventually settling in various regions of southern China [2]. These migrations occurred in five significant waves, and today, the Hakka population is spread across 85 countries and regions worldwide, with substantial populations in Jiangxi, Fujian, Guangdong, Guangxi, Hainan, Sichuan, Chongqing, Hong Kong, Macau, and Taiwan [3]. Throughout their migrations, the Hakka people developed unique traditional practices for utilizing and managing plant resources, shaped by their adaptation to diverse environments and the knowledge they acquired from both their native regions and the areas to which they migrated. These plant use and management practices have contributed to developing a distinct Hakka culture centered around food and medicine[4–6].

The Hakka medicine culture is renowned throughtout China and some other countries, playing a pivotal role in shaping traditional medicine and people’s livelihood [7]. Southeast Guangxi is a major hub for the Hakka people. Bobai County alone is home to approximately 75% (1.4 million) of the local people [8],  and Luchuan County hosting two-thirds of the local population [9]. These communities utilize the rich plant resources of Southeast Guangxi in their medicinal practices, contributing to both their healthcare and local economic development [10]. Local medicinal markets hold significant cultural and economic value, reflecting the plant diversity of the region [11]. These marketplaces support key medicinal sellers and enable rural inhabitants to sell the medicinal plants they collect, generating income to sustain their livelihoods [12]. The traditional practice of gathering and borrowing plants for medicinal use has diminished, making the local market vital for acquiring and distributing these plants, ensuring the continuity of the region's unique medicinal system [13]. Therefore, studying the local medicinal market is crucial to understanding these plants’ economic and cultural importance and advancing knowledge on their sustainable use [14].

Studies on medicinal markets have been reported in regions such as Yunnan, Sichuan, and Guizhou in China, providing valuable resources for understanding the traditional Chinese medicinal cultures of diverse ethnic groups [15, 16]. Although scholars have conducted surveys in some regions of Guangxi [17–19], a comprehensive investigation into the availability of medicinal plants in the traditional markets of the Hakka region in southeastern Guangxi remains absent. This study has conducted market surveys to document the medicinal plants traded in the local markets of Bobai County and Luchuan County in southeast Guangxi. The collected data will be compared with previous reports from similar regions of China to assess the consistency of the knowledge system and evaluate the potential for the sustainable utilization of these plants, including their use in the formulation of new medicinal products. Additionally, this study aims to protect and preserve the traditional knowledge of these medicinal plants, ensuring that valuable local practices and expertise are not lost to time and urbanization.

## Materials and methods

### Study area

Yulin City is located in the southeastern part of Guangxi Zhuang Autonomous Region, with over 7.2 million people. The majority of the population is Han Chinese. The Han dialects have three main variants: Cantonese, Min dialect, and Hakka dialect. The Hakka dialect, spoken by over 3 million people, is primarily distributed in Bobai County and Luchuan County[9]. Yulin has a mild and humid climate, characteristic of a subtropical monsoon climate, with an average temperature ranging from 22.5 to 23.2 °C and annual rainfall between 1070.6 and 1434.6 mm. In the northeast, the city is bordered by Darong Mountain, and in the southwest by Liuwan Mountain. The region’s diverse topography, including mountains, hills, valleys, terraces, and plains, is dominated by widespread hills and plateaus. Plains and basins account for 17.4% of the total area, hills, and plateaus make up 49.4%, and mountains cover 33%. This unique geographical environment and favorable climate have nurtured abundant plant resources. According to statistics, Yulin City is home to 229 families and 4343 species of vascular plants, including two species protected at the national level (Category I), eleven species protected at the national level (Category II), and eleven species designated as key protected plants in Guangxi [20]. Among the diverse wild plant resources, many medicinal plants are included. Yulin is known as the “Southern Medicinal Herb Capital” and has long relied on advantageous climate and soil resources to develop large-scale cultivation of Chinese medicinal herbs. The city is a major producer of well-known medicinal plants such as *Cinnamomum cassia*, *Illicium verum*, *Abrus pulchellus* subsp. *cantoniensis* and *Asparagus cochinchinensis* have a long history of cultivating traditional Chinese medicinal plants [21]. At present, Yulin’s medicinal herb industry has formed a large-scale, industrialized effect, with over 3.8 million acres dedicated to the cultivation of medicinal herbs, making it an essential medicinal herb distribution center in China and an influential hub for global medicinal plant trade (http://www.yulin.gov.cn/zjyl/ylgk/).

**Fig. 1:**
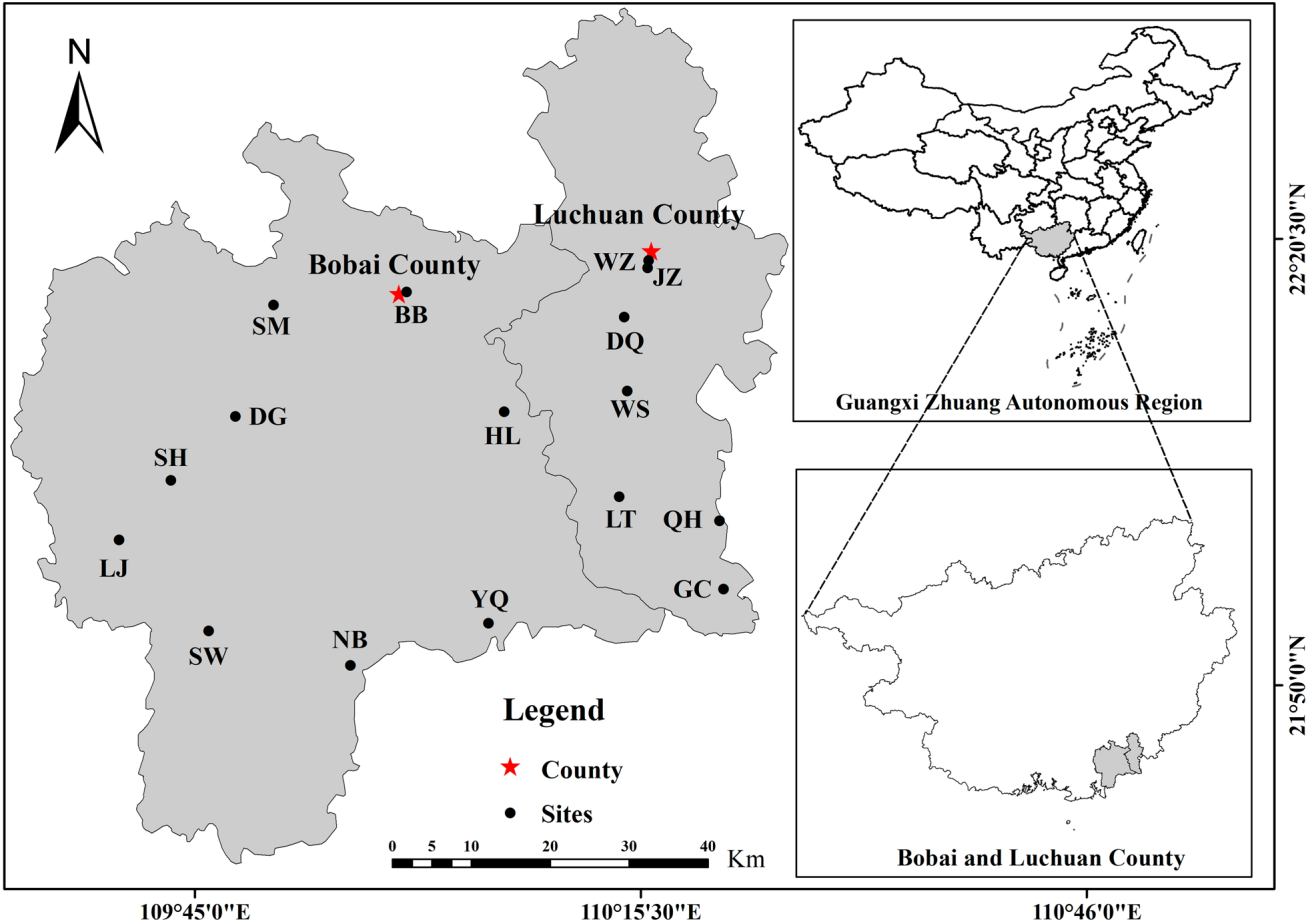
Map of the study area. (Wanzhang Market, Luchuan County; JZ: Jiuzhou Market, Luchuan County; WS: Wushi Town; DQ: Daqiao Town; LT: Liangtian Town; QH: Qinghu Town; GC: Gucheng Town; BB: Bobai Town (Dongxu Street); YQ: Yingqiao Town Market; NB: Nabu Town; HL: Huangling Town; SH: Shahe Town; SM: Shuiming Town; DG: Dungu Town; LJ: Lingjiao Town; SW: Songwang Town)

According to the literature review, combined with the results of the previous survey and the suggestions of local managers, we selected several key investigation points across Luchuan and Bobai counties, both of which are under the jurisdiction of Yulin City (Fig. 1). These sites were chosen due to their significance in local medicinal plant trade and biodiversity. The locations represent a range of both urban and rural settings, providing a comprehensive understanding of plant utilization practices within these areas (Fig. 1**)**. These investigation points cover a wide range of medicinal markets, offering valuable insights into the local trade of medicinal plants and contributing to our understanding of ecological and cultural practices surrounding medicinal plant use in these areas.

### Ethnobotanical survey

We conducted this study from August 2021 to November 2024, following the basic survey method of ethnobotany, namely the “5W + 1H” method [22]. We followed the ethical guidelines of the International Society of Ethnobiology [23] during this study period. For example, informed consent was obtained from all participants before the interviews, ensuring their voluntary participation and awareness of the study's objectives and procedures. We recorded the participant's information (name, gender, age, education, occupation) and the local name, usage, purpose, and part of the medicinal plants used. In addition, using key informants'interviews and semi-structured interviews, we visited the field to collect plant specimens from the surrounding fields, hillsides, and forests of some Hakka people who had large stalls selling herbal medicines and rich traditional knowledge of Chinese medicine. Finally, we compiled a catalog of medicinal plants and related traditional knowledge in the traditional market of southeastern Guangxi. The informants (shopkeepers) were chosen purposefully, and key informant interviews were chosen based on referrals. A total of 64 men and 42 women aged between 30 and 92 were consulted for gathering information on medicinal plants used and sold in the Hakka market. To validate the information obtained from the field, 28 key informants were consulted. The key informants were traditional herbalists, older adults with experience selling Hakka medicine, and professors who have worked in Hakka medicine for a long time.

### Plant identification and voucher collection

The voucher specimens were collected for each plant recorded in the medicinal markets of Yulin City, Guangxi. For species that were challenging to identify, follow-up investigations were carried out across different seasons to gather complete specimen materials. Each specimen was documented through high-quality photographs, capturing images of the plant, its habitat, and detailed close-ups of flowers and fruits. These voucher specimens were preserved in the herbarium of the Natural History Museum of Guangxi (NHMG). Comprehensive data were recorded for each specimen, including collection location, GPS coordinates, habitat details, phenological stages, plant characteristics, local names, scientific names, and documented uses.

Following the initial collection, plant identification was conducted through reference to trusted resources such as the *eFlora of China* (http://www.efloras.org/), the Chinese Virtual Herbarium (https://www.cvh.ac.cn/), and World Flora Online (https://www.worldfloraonline.org/). For specimens with uncertain identification, additional verification was sought from subject-matter experts in relevant botanical fields, utilizing the Chinese Virtual Herbarium for further confirmation.

The results of these field surveys, along with the identification of the collected specimens, were compiled into an ethnobotanical inventory of medicinal plants traded in the Yulin Hakka medicinal markets. This inventory includes local and scientific plant names, taxonomic classifications, and detailed information on plant uses, providing a comprehensive dataset that will support future research and applications in the region's medicinal plant trade.

### Data analysis

To ensure consistency in reporting medicinal plant usage, we categorized all diseases into 14 groups based on the International Classification of Primary Care (ICPC-2) [24], with localized adjustments informed by our research findings: physical trauma, skin diseases, heat-clearing and detoxification, digestive system diseases, nervous system diseases, respiratory system diseases, tonic conditions, urinary system diseases, ophthalmological disorders, cancer, endocrine system diseases, gynecological diseases, postpartum recovery, male-specific diseases, and others.

Relative frequency citation (RFC) was calculated to assess the consensus among the informants on the reported medical plants. It was calculated as follows:

$${\text{RFC}}\, = \,\frac{{{\text{FC}}}}{N}\,$$

RFC is the ratio of the number of respondents mentioning the use of the medical species (FC) to the total number of respondents in the survey(N) [25].

For each species, the relative importance value (RI) was calculated to understand the importance of the species in the studied area as follows;

$${\text{RI}}\,\;{ = }\,\;{\text{NM}}\,{ + }\,{\text{NT}}$$

RI is the sum of the NM and NT, which together provide a measure of the species'relative importance within the study area [26, 27]. RI for each species evaluates the species'significance in the studied area. The RI is the sum of two components: NUC and NT. NUC refers to the number of use categories attributed to a particular species, normalized by the number of use categories associated with the most versatile species in the study. Similarly, NT represents the number of different types of uses assigned to a species, normalized by the number of use types assigned to the most important taxon. This approach ensures that the RI reflects both the breadth and diversity of a species’ uses. In this study, NUC specifically corresponds to the number of therapeutic modalities (NM) assigned to a species, divided by the total number of modalities across all species.

A comparative analysis was done within the Luchan and Bobai Hakka community markets using the data from this study. Later, we also compare our results with similar studies in Hakka medicinal plants from Ganzhou [28] and Guangdong [5]. For the comparative analysis of the species with the other Hakka communities than our studies, we used only accepted names using Plants of the World Online (https://powo.science.kew.org/).In Ganzhou out of 97 reported species, only 92 were valid for the analysis, and of the 94 species 93 were validated in Guangdong. In our study, 305 species were valid for further analysis out of 308 species reported in the field. Further, we also used the Jaccard index of similarity to check the species richness recorded by our studies, along with the study of Ganzhou and Guangdong, following Jaccard [29]:

$${\text{Jaccard}}\;{\text{Index}}\;{\text{ of}}\;{\text{Similarity }}\left( {{\text{JI}}} \right)\, = \,\frac{c}{a + b - c}$$

Where a is the number of species used by residents in community A; b is the number of species used by residents in community B; and c is the number of species used by residents in both communities A and B. JI represents the similarity of plants used between communities A and B.

## Results

### General characters of the Hakka market

In the Hakka communities of Southeastern Guangxi Province of China, market days (locally termed “xu days”) are scheduled according to the lunar calendar on dates ending in 1, 4, 7, 2, 5, 8, and 3, 6, 9. On regular market days, approximately 10 stalls in the county seat sell medicinal plants, while township markets feature 3–5 such stalls. Around the Dragon Boat Festival (from the 1 st to the 5th of the fifth lunar month), the number of stalls selling medicinal plants in townships remains relatively stable. In contrast, this number increases to 15–25 stalls in the county seat. Vendors are predominantly elderly individuals over 60 years old, comprising 64 males and 42 females. Most are primarily farmers who opportunistically gather medicinal plants during market days or festivals to supplement household income. A smaller subset of vendors relies on selling herbs and practicing traditional medicine for their livelihood; these individuals typically possess extensive pharmaceutical knowledge inherited mainly from elder relatives within their clan.

**Fig. 2:**
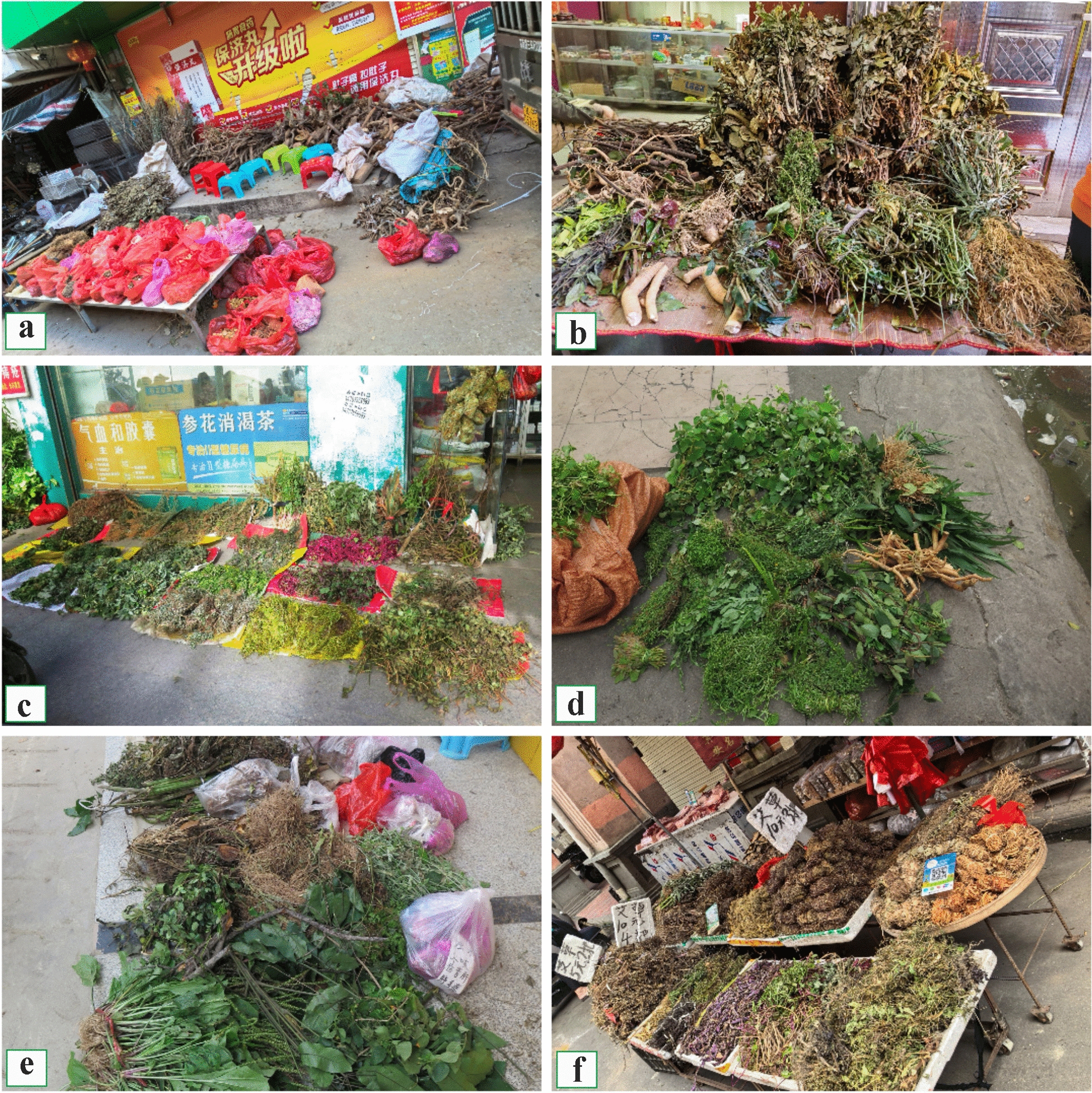
Herbs sold at local Hakka market, **a** Qinghu Town, **b** Wushi Town, **c–d** Jiuzhou Market, **e** Nabo Town, **f** Bobai Town

Medicinal plants at these markets are sold by bundle or weight (Fig. 2). Bundles typically consist of several to over a dozen herb types, priced at approximately 5–20 RMB per bundle (0.7–2.8 USD). Medicinal herbs sold by weight are primarily intended for internal use and are commonly processed into slices or sold whole. Typical examples include *Nanhaia speciosa* roots priced at 90 RMB/kg (approximately 12.6 USD/kg), whole plants of *Canscora andrographioides* at 120 RMB/kg (16.8 USD/kg), vines of *Spatholobus suberectus* at 80 RMB/kg (11.2 USD/kg), and tubers of *Curcuma phaeocaulis* at 20 RMB/kg (2.8 USD/kg).

### Medicinal plants recorded

We recorded 305 species of 103 families and 246 genera (Table 1). Among the families, Fabaceae (27 species), Asteraceae (18 species), Rubiaceae (14 species), and Lamiaceae (12 species) are the most represented. The most speciose genera include Ardisia (5 species), Ficus (4 species), and Phyllanthus (4 species), while 234 genera are either oligotypic (2 species) or monotypic (1 species). Of the 305 species recorded 94 species were globally least concern (LC), seven species were data deficit (DD), two species were nearly threatened (NT) and one was vulnerable (VU) based on the IUCN Red List of threatened species (https://www.iucnredlist.org/).

**Table 1:** List of medicinal plants reported in the field

No	Voucher IDs	Family names	Latin names	Local names	Use methods	Medicinal purposes	Life form	Sources	Medicinal parts	IUCN categories
1	LFYQ23545	Acanthaceae	*Andrographis paniculata* (Burm. f.) Wall. ex Nees	Chuan xin lian	Boil in water for drinking	Treats diarrhea, dysentery, excessive liver heat; Has heat-clearing, detoxifying, and damp-heat-eliminating effects; Prevents chicken plague	herb	wild, cultivated	Whole plant, aerial part	
2	LFYQ24196	Acanthaceae	*Clinacanthus nutans* (Burm. f.) Lindau	Bao tai teng, guo hua teng	Boil in water for washing, steam meat for consumption	Boiling in water for washing has effects of expelling fetal toxins, steaming meat for food can treat malnutrition	herb	wild, cultivated	Whole plant	
3	LFYQ23260	Acanthaceae	*Dicliptera chinensis* (L.) Juss	Gou ga teng, qing she	Boil in water for washing, with pork in soup	Treats infantile m﻿alnutrition, crying and fussing	herb	wild	Aerial part	
4	LFYQ23346	Acanthaceae	*Justicia ventricosa* Wall. ex Nees	Da bo gu	Boil in water for washing, boil in water for hot compress, soak in alcohol for hot compress	Treats bruises, fractures, dislocations, sprains; Has bone-setting, anti-inflammatory, pain-relieving, and injury-eliminating effects	herb	wild	Whole plant, aerial part	
5	LFYQ23618	Acanthaceae	*Justicia gendarussa* Burm. f	Xiao bo gu cao	Boil in water for washing, crush and apply	Treats bruises, fractures, dislocations, sprains; Has injury-eliminating, anti-inflammatory, and pain-relieving effects	herb	wild, cultivated	Aerial part	
6	LFYQ21068	Acoraceae	*Acorus calamus* L	Chang pu	Hang at the door; Boil in water for washing	Hanging at the door to expel evil; boiling in the water for washing for body pain	herb	wild, cultivated	Aerial part, whole plant	LC
7	LFYQ22349	Acoraceae	*Acorus gramineus* Aiton	Shi chang pu	Boil in water for washing	Expels evil; treats body pain	herb	wild, cultivated	Aerial part, whole plant	LC
8	LFYQ22007	Altingiaceae	*Liquidambar formosana* Hance	San cha feng, lu lu tong	Branches and leaves boiled in water for washing, fruit boiled in water for drinking	Boiling in water for washing expels wind-cold, expels wind, treats post-partum wind syndrome, reduces swelling, promotes blood circulation, relieves pain, treats body and foot pain; Fruit boiled in water for drinking treats wind, promotes blood flow, and treats cramps	tree	wild	Branches, fruit	LC
9	LYFQ21203	Amaranthaceae	*Achyranthes aspera* L	Ma bian cao, gou yao cao	Boil in water for drinking	Treats renal calculi , urinary problems, bone hyperplasia; Has heat-clearing, detoxifying, and anti-inflammatory effects	herb	wild	Aerial part	
10	LFYQ22212	Amaranthaceae	*Alternanthera brasiliana* (L.) Kuntze	Xiao chang feng	Boil in water for drinking	Treats dysmenorrhea; Has heat-clearing and detoxifying effects	herb	wild	Aerial part	
11	LFYQ24209	Amaranthaceae	*Alternanthera philoxeroides* (Mart.) Griseb	Kong xin lian zi cao	Boil in water for drinking	Treats sore throat and toothache	herb	wild	Whole plant	
12	LFYQ22069	Amaranthaceae	*Amaranthus spinosus* L	Le xian cai, Hong le xian cai	Boil in water for drinking, boil in water for washing	Boiling in water for drinking treats gastrointestinal damp-heat, enteritis, high fever, yellow urine, heat-toxicity; with anti-diarrhea, cooling blood, and blood-toning effects; boiling in water for washing relieves itching	herb	wild	Whole plant, roots	
13	LYFQ21201	Amaranthaceae	*Celosia argentea* L	Qing xiang	Boil in water for washing, with pig's nose in cooking, boil in water for drinking	With pig's nose in cooking treats nosebleeds; Boiling in water for drinking has heat-clearing and detoxifying effects, boiling for washing has anti-inflammatory effects	herb	wild	Aerial part	LC
14	LFYQ22606	Amaranthaceae	*Cyathula prostrata* (L.) Blume	Di dan	Boil in water for washing	Treats fetal toxins	herb	wild	Whole plant	
15	LFYQ21112	Anacardiaceae	*Mangifera indica* L	Mang guo	Boil in water for drinking	Treats liver diseases, has anti-inflammatory and detoxifying effects	tree	cultivated	Branches	DD
16	LFYQ23211	Anacardiaceae	*Rhus chinensis* Mill	Yan fu mu	Boil in water for washing	Boiling in water for washing relieves itching and sweating, treats cold sweats and excessive sweating	tree	wild	Branches	LC
17	LFYQ22240	Annonaceae	*Desmos chinensis* Lour	Ji zhua feng	Boil in water for washing, boil in water for drinking	Expels wind-cold, expels wind, treats postpartum cold, used for postpartum wind syndrome and swelling	shrub	wild	Branches	
18	LFYQ23050	Annonaceae	*Fissistigma polyanthum* (Hook. f. & Thomson) Merr	Hei gu teng	Boil in water for drinking	Treats rheumatic bone pain, internal wind-damp	shrub	wild	Stem	
19	LFYQ 21056	Annonaceae	*Uvaria littoralis* (Blume) Blume	Jiu bing po	Boil in water for washing	Has blood-moving, wind-expelling, and postpartum wind syndrome, swelling-reducing, and pain-relieving effects	shrub	wild	Branches	
20	LFYQ24369	Annonaceae	*Uvaria grandiflora* Roxb.ex Hornem	Shan jiao	Boil in water for washing	Used for postpartum wind syndrome, has swelling-reducing and hemostatic effects	shrub	wild	Branches	
21	LFYQ22065	Apiaceae	*Centella asiatica* (L.) Urb	Lei gong gen, da yang lei gong gen	Boil in water	Has heat-clearing and detoxifying effects	herb	wild	Whole plant	LC
22	LFYQ23498	Apocynaceae	*Cynanchum graphistemmatoides* Liede & Khanum	Po lang, da nai cao	With pig's feet in soup, boil in water for drinking	Treats numbness in hands and feet, cramps, hand pain, has postpartum lactation-promoting effects	liana	wild	Vine leaves	
23	LFYQ22464	Apocynaceae	*Dischidia chinensis* Champ. ex Benth	Ma lou hai, fei bie	With pork in soup	Expels m﻿alnutrition, treats newborn skin folds, has saliva-promoting and quenching thirst effects	herb	wild	Whole plant	
24	LYFQ21175	Apocynaceae	*Strophanthus divaricatus* Hook. & Arn	Yang jiao niu	Boil in water for drinking	Treats internal heat sores, iron injuries	shrub	wild	Branches	
25	LFYQ23044	Apocynaceae	*Urceola micrantha* (Wall. ex G. Don) D. J. Middleton	Teng du zhong	Boil in water for drinking	Has kidney-toning effects	liana	wild	Stem	
26	LFYQ23366	Apocynaceae	*Urceola rosea* (Hook. & Arn.) D.J.Middleton	Hong bei suan, hong bei jiang jun teng	Boil in water for washing	Treats wound redness, scabs, sha, foot ulcers, soreness, has swelling-reducing and pain-relieving effects	liana	wild	Vine leaves	
27	LFYQ23586	Apocynaceae	*Vincetoxicum pycnostelma* Kitag	Liao diao zhu	Soak in alcohol for drinking	Treats bruises and sprains	herb	wild, purchased	Roots	
28	LFYQ23581	Aquifoliaceae	*Ilex asprella* Champ. ex Benth	Cheng xing mu gen	Boil in water for drinking, slice and suck	Treats sore throat and pharyngitis; Has anti-inflammatory, heat-clearing, detoxifying, saliva-promoting and thirst-quenching effects	tree	wild, cultivated	Roots	
29	LFYQ22348	Aquifoliaceae	*Ilex rotunda* Thunb	Rong dan mu	Boil in water for drinking	Treats heat sensation, infantile malnutrition, sore throat, has fire-reducing, liver-cooling, and lung-cooling effects	tree	wild	Bark, roots	LC
30	LFYQ22703	Aquifoliaceae	*Ilex pubescens* Hook. & Arn	Mao dong qing	Boil in water for drinking	Treats neuralgia, can promote blood circulation	tree	wild	Roots, stem	
31	LFYQ24204	Araceae	*Alocasia odora* (G.Lodd.) Spach	Lang du	Slice and heat for external application	Treats shingles	herb	wild	Stem	LC
32	LFYQ22313	Araceae	*Amorphophallus konjac* K. Koch	Fan yu	Cook with chicken	Has fire-reducing and blood-toning effects; Can prevent recurrence of disease	herb	cultivated	Tuber	
33	LFYQ22684	Araceae	*Epipremnum pinnatum* (L.) Engl	Pa qiang feng	Boil in water for washing, boil in water for drinking	Treats rheumatic bone pain	liana	wild, cultivated	Vine leaves	
34	LYFQ21283	Araceae	*Lasia spinosa* (L.) Thwaites	Shui le gou	Boil in water for drinking	Treats liver heat, gastrointestinal heat, cancer	herb	wild	Roots, aerial part	LC
35	LFYQ22088	Araliaceae	*Dendropanax proteus* (Champ. ex Benth.) Benth	Feng he gui	Soak in alcohol for drinking, boil in water for drinking	Expels wind and dampness, treats wind-paralysis	shrub	wild, cultivated	Branches	
36	LFYQ22700	Araliaceae	*Heptapleurum heptaphyllum* (L.) Y. F. Deng	Ya jiao mu, ya ma jiao	Boil in water for washing	Has wind-damp expelling effects	tree	wild	Branches	LC
37	LFYQ22016	Araliaceae	*Hydrocotyle sibthorpioides* Lam	Xi ye lei gong gen	Boil in water for drinking	Has cooling, fire-reducing, and anti-inflammatory effects, treats tooth pain	herb	wild	Whole plant	LC
38	LFYQ23613	Aristolochiaceae	*Aristolochia fordiana* Hemsl	Tong cheng hu	Boil in water for drinking	Relieve pain; treats body pain, bruises, and snakebites	liana	wild, purchased	Roots	
39	LFYQ22678	Asparagaceae	*Agave americana* L	Jin bian lian, jin bian lan	Boil in water for drinking; with pig's lung in soup	Treats lung heat, cough; Has lung-cooling and antitussive effects	herb	cultivated	Leaves	LC
40	LFYQ21299	Asparagaceae	*Cordyline fruticosa* (L.) A. Chev	Hong tie shu, tie shu ye	Boil in water for drinking, leaves with pork in soup, boil in water for washing	Root boiled in water for drinking treats diarrhea; Leaves boiled in water for drinking treats diarrhea, bruises,hematemesis, hematochezia, nosebleeds, dysmenorrhea, prostatitis, and gastrointestinal heat; Has blood-toning and hemostatic effects; Leaves with pork in soup treats bruises; Branches and leaves boiled in water treats hemorrhoids	shrub	cultivated	Roots, leaves, branches	LC
41	LFYQ22275	Asparagaceae	*Dracaena elliptica* Thunb. & Dalm	Zhu mu shen	Boil in water for drinking	Treats heart disease, pneumonia, hepatitis, heart pain, wind-paralysis; Has lung-clearing, injury-eliminating, and antitussive effects	shrub	wild	Roots, branches	LC
42	LFYQ22479	Asphodelaceae	*Aloe vera* (L.) Burm. f	Lu hui	Use raw for external application	Treats burns; has anti-itching effects	herb	cultivated	Leaves	
43	LFYQ22718	Asphodelaceae	*Dianella ensifolia* (L.) Redouté	Niu shen, gao jian huang	Boil in water for washing	Expels heat toxins and reduces jaundice	herb	wild	Whole plant	
44	LFYQ23012	Asteraceae	*Ageratum conyzoides* L	Chou cao, bai hua chou cao	Boil in water for washing, crush and apply externally, boil in water for drinking	Boil aerial parts in water and used in washing can expel wind and stop itching; crushed tender leaves applied externally has hemostatic effect; drinking the boiled water treats high fever and has antitussive effects	herb	wild	Aerial part, leaves	
45	LFYQ21028	Asteraceae	*Artemisia lactiflora* Wall. ex DC	Tian cai, hong yao ai	Boil in water for drinking	Treats irregular menstruation; Has lung-moisturizing, cough-relieving, and phlegm-expelling effects	herb	cultivated	Whole plant, aerial part	
46	LFYQ23149	Asteraceae	*Artemisia indica* Willd	Ai	Boil in water for washing, boil in water for drinking, with pig's blood in cooking	Boiling in water for washing can relieve itching, reduce swelling, expel wind, and expel dampness; Treats bone pain, used for postpartum wind syndrome , swelling reduction, and pain relief; Boilling in water for drinking or with pig's blood for eating have hemostatic effect and expels wind	herb	wild	Aerial part	
47	LFYQ21008	Asteraceae	*Aster indicus* L	Ju hua	Boil in water for drinking	Can reduce internal heat; Treats red eyes	herb	wild	Whole plant or flowers	
48	LFYQ24153	Asteraceae	*Bidens alba* (L.) DC	Yi bao zhen	Boil in water for washing, boil in water for drinking	Boiling in water for washing clears heat and relieves itching; Tender leaves boiled in water for drinking clears heat and detoxify	herb	wild	Aerial part, young leaves	
49	LFYQ21133	Asteraceae	*Blumea balsamifera* (L.) DC	Da feng ai	Boil in water for washing	Used for postpartum wind syndrome and swelling reduction; Has wind-expelling, dampness-expelling, and heat-clearing effects	herb	wild	Branches, whole plant	LC
50	LYFQ21150	Asteraceae	*Blumea riparia* DC	Bai hua jiu li ming	Boil in water for drinking, boil in water for washing	Boiling in water for drinking clears the liver and improves eyesight; Boiling in water for washing relieves itching and treats skin diseases	liana	wild	Vine leaves	
51	LFYQ23098	Asteraceae	*Centipeda minima* (L.) A. Braun & Asch	E bu shi cao	Crush and apply inside the nose, boil in water for drinking	Treats rhinitis	herb	wild	Whole plant	LC
52	LYFQ21214	Asteraceae	*Crassocephalum crepidioides* (Benth.) S. Moore	Pu gong ying	Tender leaves stir-fried for consumption, aerial parts boiled in water for drinking	Treats uterine cancer and toxic sores; Has heat-clearing and detoxifying effects	herb	wild	Aerial part, young leaves	
53	LFYQ21122	Asteraceae	*Inula cappa* (Buch.-Ham. ex D.Don) DC	Bai bei feng	With meat and bones in soup, boil in water for drinking	Treats stomachache , bruises, irregular menstruation	shrub	wild	Branches	
54	LFYQ22172	Asteraceae	*Eclipta prostrata* (L.) L	wu mo cao	Boil in water for drinking, crush and apply, boil in water for washing	Crushed tender leaves applied to treat oral ulcers; Boiling the whole plant in water for drinking has anti-inflammatory and wind-expelling effects; Boiling the whole plant in water for drinking has heat-clearing, detoxifying, and cough-relieving effects	herb	wild	Whole plant, young leaves	LC
55	LFYQ23361	Asteraceae	*Elephantopus scaber* L	Di dan tou	Boil in water for drinking	Has heat-clearing, detoxifying, and liver-cooling effects	herb	wild	Whole plant, rhizome	
56	LFYQ23517	Asteraceae	*Emilia sonchifolia* (L.) DC	Yi dian hong	Boil in water for drinking, crush and apply	Crushed application treats red eyes, has anti-inflammatory, heat-clearing, and detoxifying effects; Boiling in water for drinking clears heat and detoxifies	herb	wild	Aerial part, whole plant	
57	LFYQ23554	Asteraceae	*Gymnanthemum amygdalinum* (Delile) Sch.Bip	Fei zhou mu ye	Boil in water for drinking	Has heat-clearing and detoxifying effects	shrub	wild	Branches	
58	LFYQ23450	Asteraceae	*Senecio scandens* (L.) Buch.-Ham	Jiu li ming	Boil in water for washing, boil in water for drinking	Boiling in water for washing treats itching; Boiling in water for drinking clears the liver and brightens the eyes	herb	wild	Aerial part	LC
59	LFYQ22582	Asteraceae	*Stevia rebaudiana* Bertoni	Shan mi cao, bing tang cao	Boil in water for drinking	Has heat-clearing and detoxifying effects	herb	purchased	Whole plant	
60	LYFQ21272	Asteraceae	*Taraxacum mongolicum* Hand.-Mazz	Pu gong ying	Boil in water for drinking	Treats sore , has detoxifying and damp-expelling effects	herb	wild	Whole plant, leaves	
61	LFYQ22651	Asteraceae	*Vernonia cumingiana* Benth	Guo shan long	Boil in water for drinking	Treats sore throat, tonsillitis, sore throat, diarrhea, stomach pain, vomiting, has pain-relieving and anti-diarrheal effects	liana	wild	Vine leaves	
62	LFYQ21002	Basellaceae	*Basella alba* L	Hong lan cai zi	Boil in water for washing, boil in water for drinking	Has detoxifying, heat-clearing, and heat-toxicity-expelling effects	herb	cultivated	Aerial part	
63	LFYQ22144	Berberidaceae	*Berberis bealei* Fortune	Shi da gong lao	Boil in water for drinking	Treats hepatitis, cirrhosis	shrub	wild	Stem	
64	LFYQ24346	Bignoniaceae	*Markhamia stipulata* (Wall.) Seem. ex K. Schum	Mao wei shu	Boil in water for washing, boil in water for drinking	Boiling in water for washing treats sore , boiling in water for drinking treats hepatitis	tree	wild	Stem	LC
65	LFYQ21121	Boraginaceae	*Cordia dichotoma* G. Forst	Ba pi mu	Boil in water for washing, steam meat for consumption	Boiling in water for washing expels fetal toxins, steaming meat can expel m﻿alnutrition	tree	wild	Branches	LC
66	LFYQ22687	Calophyllaceae	*Calophyllum membranaceum* Gardner & Champ	Heng jing xi, bi zi wang, da shan jian	Boil in water for drinking, with meat and bones in soup, soak in alcohol for drinking	Treats bruises, bone pain, hand pain, joint pain, lumbago, internal injuries, and renal calculi; Has swelling-reducing effects; Boiling in water can treat pig plague	tree	Wild	Stem, branches, roots	LC
67	LFYQ23126	Campanulaceae	*Lobelia chinensis* Lour	Ban bian lian	Boil in water for drinking	Treats snake venom, has heat-clearing and detoxifying effects	herb	wild	Whole plant	
68	LFYQ23445	Capparaceae	*Capparis acutifolia* Sweet	Du xing qian li	Boil in water for washing	Treats snakebites	shrub	wild	Branches, stem	
69	LFYQ24025	Capparaceae	*Capparis versicolor* Griff	Qu tou ji	Boil in water for drinking	Treats epilepsy	liana	wild	Fruit	
70	LFYQ23638	Caprifoliaceae	*Lonicera confusa* (Sweet) DC	Jin yin hua	Boil in water for drinking	Treats stomach heat, has heat-clearing and detoxifying effects	liana	wild	Vine leaves or flowers	
71	LFYQ22719	Chloranthaceae	*Sarcandra glabra* (Thunb.) Nakai	Jiu jie feng, shan ji cha	Boil in water for drinking, boil in water for washing	Boiling in water for drinking treats sore throat, joint pain, paralysis, has bone-setting, stomach-strengthening, and anti-tumor effects; Boiling in water for washing treats rheumatic bone pain, post-partum recovery, and bruises, has blood-moving, blood-promoting, and bone-setting effects	shrub	wild	Whole plant, branches	
72	LFYQ23177	Cibotiaceae	*Cibotium barometz* (L.) J. Sm	Huang gou tou	Boil in water for drinking	Treats rheumatic bone pain, kidney deficiency; Has effects of promoting circulation, expelling injury accumulation, tonifying body, toning kidneys, and has hemostatic effect	herb	wild	Rhizome	
73	LFYQ23460	Clusiaceae	*Garcinia oblongifolia* Champ. ex Benth	Huang ya mu	Boil in water for drinking	Has heat-clearing and detoxifying effects	tree	wild	Fruit peel	LC
74	LFYQ22680	Commelinaceae	*Callisia repens* (Jacq.) L	Zhu ke cai	Boil in water for drinking	Treats urethritis, cystitis; Has diuretic effects	herb	Wild	Whole plant	
75	LFYQ23151	Commelinaceae	*Commelina diffusa* Burm. f	Piao wang, piao yao	Boil in water for drinking, boil in water for washing	Boiling in water for drinking treats hepatitis; Boiling in water for washing treats bruises and jaundice	herb	wild	Whole plant	LC
76	LFYQ23494	Convolvulaceae	*Argyreia acuta* Lour	Bai mian shui ji	Boil in water for drinking	Treats pneumonia; with cough-relieving, phlegm-expelling, and lung-moisturizing effects	shrub	wild	Branches	
77	LFYQ22175	Convolvulaceae	*Cuscuta chinensis* Lam	Huang teng	Boil in water for washing	Has jaundice-removing effects	herb	wild	Aerial part	
78	LFYQ24205	Cucurbitaceae	*Gynostemma pentaphyllum* (Thunb.) Makino	Jiao gu lan	Boil in water for drinking	Has heat-clearing and detoxifying effects	liana	wild	Aerial part	
79	LFYQ22572	Cucurbitaceae	*Momordica charantia* L	Ku gua tou	Boil in water for drinking, boil in water for hot compress	Treats body pain, has cooling effects	herb	cultivated	Roots	
80	LFYQ23380	Cucurbitaceae	*Momordica cochinchinensis* (Lour.) Spreng	Mu bie zi	Root boiled in water for washing, fruit made into cake, seeds boiled in water for drinking	Root boiled in water for washing has swelling-reducing and detoxifying effects; Fruit made into cake treats infantile m﻿alnutrition, injury accumulation, and treats children's sore ; Seeds boiled in water for drinking treats gastrointestinal inflammation, with injury-eliminating, swelling-reducing, and pain-relieving effects	liana	wild, cultivated	Roots, fruit, seeds	
81	LFYQ22583	Cucurbitaceae	*Siraitia grosvenorii* (Swingle) C. Jeffrey ex A. M. Lu & Zhi Y. Zhang	Luo han guo	Boil in water for drinking	Has heat-clearing and detoxifying effects	herb	purchased	Fruit	
82	LFYQ 23356	Cupressaceae	*Platycladus orientalis* (L.) Franco	Bai mu	Boil in water for drinking	Treats nosebleeds, wound bleeding, constipation, and neurasthenia, has hemostatic and cough-relieving effects	tree	wild	Branches	NT
83	LFYQ24027	Cyperaceae	*Cyperus rotundus* L	Cong cao shu	Boil in water for drinking	Treats liver and lung heat, flatulence	herb	wild	Whole plant	LC
84	LYFQ21167	Cyperaceae	*Cyperus mindorensis* (Steud.) Huygh Camus	Zei cao	Boil in water for hot compress, boil in water for washing	Treats bruises	herb	wild	Whole plant	
85	LFYQ22258	Daphniphyllaceae	*Daphniphyllum calycinum* Benth	Niu er feng	Boil in water for washing	Treats postpartum wind syndrome, with wind-damp-expelling effects	shrub	wild	Branches	LC
86	LYFQ21186	Dilleniaceae	*Tetracera sarmentosa* (L.) Vahl	Yang sha teng	Boil in water for drinking	Boiling in water for drinking treats colds, stroke, infantile m﻿alnutrition, with anti-diarrheal effects	liana	wild	Vine leaves	
87	LFYQ22738	Dioscoreaceae	*Dioscorea fordii* Prain & Burkill	Ye sheng huai shan, ye shan yao	Boil in water for drinking, with meat and bones in soup	Has effects of tonifying spleen and stomach, transforming dampness	liana	wild	Roots	
88	LYFQ21189	Equisetaceae	*Equisetum hyemale* L	Bi ta cao	Boil in water for drinking	Treats cataracts, corneal inflammation, liver and gallbladder heat; Has heat-clearing and detoxifying effects	herb	wild	Aerial part	LC
89	LFYQ23495	Eriocaulaceae	*Eriocaulon sexangulare* L	Niu cao, yu yan jing	Boil in water for drinking, add meat and steam for consumption	Boiling in water for drinking treats eye heating and treats the liver problems; steaming with meat for food treats infantile malnutrition	herb	wild	Aerial part	
90	LFYQ22012	Euphorbiaceae	*Alchornea trewioides* Müll. Arg	Hong mao ding	Boil in water for washing	Has heat-clearing and heat-toxicity-expelling effects	shrub	wild	Branches	
91	LFYQ22391	Euphorbiaceae	*Euphorbia hirta* L	Fei yang cao	Boil in water for washing	Has anti-itching effects	herb	wild	Whole plant	
92	LFYQ22201	Euphorbiaceae	*Mallotus barbatus* Müll. Arg	Hou pi zhan	Boil in water for washing	Treats urticaria, relieves itching, expels heat toxins	tree	wild	Branches	LC
93	LFYQ22546	Euphorbiaceae	*Mallotus philippensis* (Lam.) Müll.Arg	Cu kang chai	Boil in water for drinking	Has anti-diarrheal effects	shrub	wild	Branches	LC
94	LFYQ24130	Euphorbiaceae	*Triadica sebifera* (L.) Small	Wu jiu	Boil in water for washing	Has anti-itching effects	tree	wild	Branches	LC
95	LYFQ21245	Euphorbiaceae	*Triadica cochinchinensis* Lour	Shan wu jiu	Boil in water for washing	Treats sprains, has anti-itching effects	tree	wild	Branches	LC
96	LFYQ23200	Fabaceae	*Abrus melanospermus* subsp. *melanospermus*	Ji gu cao	Boil in water for drinking	Treats hepatitis B (types D and C), yellow urine, renal calculi, and hepatitis; Has heat-clearing, detoxifying, lung-moisturizing, and liver-cooling effects	liana	wild, cultivated	Whole plant	
97	LFYQ23532	Fabaceae	*Abrus precatorius* L	A gong yan teng	Boil in water for drinking	Has cooling and detoxifying effects	liana	wild, cultivated	Whole plant	
98	LFYQ24161	Fabaceae	*Bauhinia* × *blakeana* Dunn	Ma shu ye	Boil in water for washing	Has anti-itching and wind-expelling effects	tree	cultivated	Branches	
99	LFYQ23365	Fabaceae	*Biancaea sappan* (L.) Tod	Su mu	Boil in water for drinking, boil in water for washing	Boiling in water for drinking and washing promotes blood circulation, reduces swelling and pain, and promotes blood circulation; Treats bruises, dysmenorrhea, qi and blood deficiency, and abdominal pain	tree	wild	Stem	LC
100	LFYQ24152	Fabaceae	*Cajanus cajan* (L.) Millsp.	Mu dou	Boil in water for washing	Treats chickenpox	shrub	Wild	Branches	
101	LFYQ22694	Fabaceae	*Canavalia rosea* (Sw.) DC	Tou yu teng	Boil in water for washing	Has anti-itching and antibacterial effects; Treats body itching	liana	Wild	Vine leaves	LC
102	LFYQ22319	Fabaceae	*Chamaecrista mimosoides* (L.) Greene	Ruan gan cao, shan sao ba	Boil in water	Treats liver and gallbladder heat, hepatitis B (types D and C), hepatitis, cirrhosis	herb	wild	Aerial part	LC
103	LFYQ24214	Fabaceae	*Cheniella corymbosa* (Roxb. ex DC.) R.Clark & Mackinder	Fei yang teng	Boil in water for washing	Treats body itching	liana	wild	Vine leaves	
104	LFYQ23306	Fabaceae	*Crotalaria sericea* Burm.f	Zhu shi dou	Boil in water for drinking	Treats hepatitis	herb	wild	Aerial part	LC
105	LFYQ23519	Fabaceae	*Entada phaseoloides* (L.) Merr	Guo jiang long	Boil in water for drinking	Has effects of soothing tendons and promoting circulation, reducing swelling, relieving pain, expelling wind, and removing dampness; Treats rheumatic bone pain, lumbago, hand and foot pain, stroke	liana	wild	Stem	
106	LFYQ22196	Fabaceae	*Flemingia macrophylla* (Willd.) Kuntze ex Merr	Qian jin ba	Boil in water for drinking	Has effects of strengthening tendons, tonifying bones, tonifying heart, and tonifying kidneys	shrub	wild	Roots	
107	LFYQ22045	Fabaceae	*Flemingia prostrata* Roxb. f. ex Roxb	Qian jin bo	Boil in water for drinking, with bones in soup	Treats lumbago, has effects of soothing tendons and promoting circulation	shrub	wild	Roots	
108	LFYQ23338	Fabaceae	*Fordia cauliflora* Hemsl	Tie luo san	Boil in water for drinking, boil in water for washing, with bones in soup	Boiling in water for washing treats bruises, has effects of dispersing joints, reducing swelling, and relieving pain; Boiling in water for drinking promotes qi; With bones in soup, treats lumbago and has pain-relieving effects	shrub	cultivated	Roots	LC
109	LFYQ24208	Fabaceae	*Gleditsia sinensis* Lam	Lu jiao ci	Boil in water for drinking, with pig's heart in soup, make into powder for ointment	Boiling in water for drinking or using pig's heart in soup treats heart pain and wound pain; Powdered ointment treats abscesses	tree	wild, purchased	Thorns	LC
110	LFYQ24217	Fabaceae	*Glycine max* (L.) Merr	Wu dou, huo dou	Boil in water for drinking, with bones in soup, cook in porridge	Has heat-clearing and detoxifying effects	herb	cultivated	Seeds	
111	LFYQ22607	Fabaceae	*Grona heterocarpos* (L.) H. Ohashi & K. Ohashi	Jia mu dou, jia fan dou	Boil in water for washing, boil in water for drinking	Boiling in water for washing has anti-itching, heat-clearing and detoxifying effects; Boiling in water for drinking clears heat and detoxifies	shrub	wild	Branches	
112	LFYQ22656	Fabaceae	*Grona styracifolia (Osbeck)* H. Ohashi & K. Ohashi	Jin qian cao	Boil in water for drinking, boil in water for washing	Boiling in water for drinking treats sore throat, renal calculi, gall calculi, bladder calculi, and hepatitis; Has anti-inflammatory, heat-clearing, detoxifying, diuretic, liver-cooling, and gastrointestinal cooling effects; Boiling in water for washing treats children's skin diseases	herb	wild	Aerial part	
113	LFYQ22083	Fabaceae	*Kummerowia striata* (Thunb.) Schindl	Ren zi cao	Boil in water for drinking	Treats liver diseases	herb	wild	Whole plant	
114	LFYQ23030	Fabaceae	*Nanhaia speciosa* (Champ. ex Benth.) J. Compton & Schrire	Niu da li	With bones in soup, soak in alcohol for 3 years for drinking, boil in water for drinking	Soaking in alcohol for drinking strengthens the waist, tonifies the kidneys, strengthens bones; With bones in soup, tonifies kidneys, reduces swelling, and treats bone pain; Boiling in water for drinking relieves pain, hemostasis, and treats internal injuries, hematemesis, and sprains	liana	cultivated	Roots	
115	LFYQ22290	Fabaceae	*Phanera championii* Benth	Jiu long teng	Boil in water for drinking, boil in water for washing	Treats rheumatic bone pain, numbness in hands and feet, bruises, and pain; Boiling in water for washing can stop itching and treat skin diseases	liana	wild	Stem, roots	
116	LYFQ21191	Fabaceae	*Phyllodium elegans* (Lour.) Desv	Zhi qian, jia mu dou	Boil in water for washing	Treats infantile m﻿alnutrition, neonatal skin diseases, has heat-clearing and detoxifying effects	shrub	wild	Branches	LC
117	LFYQ22621	Fabaceae	*Pueraria montana* var. *lobata* (Ohwi) Maesen & S. M. Almeida	Ge gen	With bones in soup, boil in water for drinking	With bones in soup, treats hypertension; Boiling in water for drinking clears fire and reduces heat	liana	cultivated	Tuberous root	
118	LFYQ22421	Fabaceae	*Spatholobus suberectus* Dunn	Ji xue teng	Boil in water for washing, boil in water for drinking, with bones in soup, soak in alcohol for drinking	Boiling in water for drinking promotes blood circulation, tonifies the blood, relieves pain, treats irregular menstruation , breathing difficulty, bruises, foot pain, numbness in hands and feet; Soaking in alcohol for drinking or with bones in soup tonifies blood, promotes blood circulation; Boiling in water for washing relieves swelling, pain, and treats bruises	liana	wild, cultivated	Stem	
119	LFYQ23533	Fabaceae	*Spatholobus harmandii* Gagnep	Li zhi ye ji xue teng	Boil in water for drinking	Tonifies blood	liana	wild, cultivated	Stem	
120	LFYQ22163	Fabaceae	*Tadehagi triquetrum* (L.) H. Ohashi	Hu lu cha	Boil in water for drinking, boil in water for washing	Boiling in water for drinking clears heat and detoxifies; Boiling in water for washing relieves itching, expels heat toxins, and expels wind	shrub	wild	Aerial part	
121	LFYQ22339	Fabaceae	*Uraria crinita* (L.) Desv. ex DC	Lao hu wei	Boil in water for drinking	Treats lumbago, urethritis, cystitis, has kidney-toning, anti-inflammatory, hemostatic, and pain-relieving effects	shrub	wild, cultivated	Aerial part, whole plant	
122	LFYQ22158	Fabaceae	*Uraria lagopodioides* (L.) Desv. ex DC	Lao hu wei	Boil in water for drinking	Treats lumbago, has blood-toning and kidney-toning effects	herb	wild, cultivated	Aerial part	
123	LFYQ24227	Fagaceae	*Lithocarpus pachylepis* A. Camus	Feng liu guo	Boil in water for drinking	Has kidney-toning, blood-toning, and blood-promoting effects	tree	purchased	Fruit	VU
124	LFYQ22720	Gentianaceae	*Canscora andrographioides* Griff. ex C. B. Clarke	Ji xin huang, si fang cao, shen xian cao	Soak in alcohol for drinking, boil in water for drinking	Treats liver and gallbladder heat; Has heat-clearing and detoxifying effects	herb	wild, purchased	Whole plant	
125	LFYQ23601	Gnetaceae	*Gnetum parvifolium* (Warb.) C. Y. Cheng ex Chun	Ma gu feng	Boil in water for washing	Treats rheumatism, numbness, and psoriasis	liana	wild	Stem	LC
126	LFYQ22282	Hypericaceae	*Cratoxylum cochinchinense* (Lour.) Blume	Huang ya ding, huang ya mu	Boil in water for drinking, boil in water for washing	Boiling in water for drinking treats stomachache and diarrhea; Root, stem, and leaves boiled in water for drinking treats hepatitis; Branches and leaves boiled in water for washing treat jaundice in children	shrub	wild	Roots, branches, fruit	LC
127	LFYQ22407	Hypericaceae	*Hypericum japonicum* Thunb	Tian ji huang, huang hua wu	Boil in water for drinking	Has liver-cooling effects, treats liver heat, hepatitis, and gastrointestinal heat	herb	wild	Whole plant	
128	LFYQ22309	Hypoxidaceae	*Curculigo orchioides* Gaertn	Du jiao si mao	Boil in water for drinking	Has kidney-toning effects	herb	wild, cultivated	Whole plant	
129	LFYQ23138	Iridaceae	*Iris domestica* (L.) Goldblatt & Mabb	Gao jian zi	Boil in water for drinking, boil in water for washing	Boiling in water for drinking treats yellow urine and liver heat; Boiling in water for washing expels jaundice, relieves itching, and treats skin rashes	herb	wild	Whole plant or aerial part	
130	LFYQ23194	Juglandaceae	*Engelhardia roxburghiana* Lindl	Shan huang ju	Boil in water for drinking, stems, leaves, and bark dried for tea, with chicken in soup	Boiling in water for drinking tea has heat-clearing, detoxifying, and weight-loss effects; With chicken in soup treats tooth pain, has fire-reducing effects	tree	wild	Branches, leaves, bark	LC
131	LFYQ23136	Juncaceae	*Juncus effusus* L	Deng xin cao	Boil in water for drinking	Has heat-clearing, detoxifying, and lung-moisturizing effects	herb	wild, cultivated	Aerial part, whole plant	LC
132	LFYQ23137	Juncaceae	*Juncus inflexus* L	Deng xin cao	Boil in water for drinking	Treats dryness and heat; Has heat-clearing, detoxifying, and heart-fire-reducing effects	herb	wild, cultivated	Whole plant, aerial part	LC
133	LYFQ21288	Lamiaceae	*Clerodendrum bungei* Steud	Chou mu dan	Boil in water for washing	Has wind-expelling effects	shrub	wild	Branches	LC
134	LFYQ22449	Lamiaceae	*Clerodendrum cyrtophyllum* Turcz	Yang wei qing	Boil in water for washing	Has anti-itching effects	shrub	wild	Branches	LC
135	LFYQ22580	Lamiaceae	*Clerodendrum fortunatum* L	Gui deng long, hong ya gong qing	Boil in water for drinking	Has heat-clearing and detoxifying effects	shrub	wild	Branches	LC
136	LFYQ23607	Lamiaceae	*Glechoma longituba* (Nakai) Kuprian	Tou gu xiao, bo gu xiao	Boil in water for hot compress, boil in water for washing	Treats bruises, has effects of injury elimination, anti-inflammatory, and pain-relieving	herb	wild	Whole plant	
137	LFYQ23631	Lamiaceae	*Isodon ternifolius* (D. Don) Kudô	San jie mei	Boil in water for drinking	Treats severe colds, liver cancer, hepatitis; Has detoxifying and anti-inflammatory effects	herb	wild, purchased	Aerial part	
138	LFYQ24083	Lamiaceae	*Leonurus japonicus* Houtt	Yi mu cao	Boil in water for drinking, boil in water for washing, with eggs, pork small stomach in cooking	Boiling in water for drinking cools the blood, treats dysmenorrhea, irregular menstruation, and infertility; Boiling in water for washing treats irregular menstruation; With eggs and pork small stomach, treats stomach pain, used for postpartum wind syndrome and pain relief	herb	wild	Whole plant, aerial part	
139	LFYQ23010	Lamiaceae	*Mentha canadensis* L	Bo he, si fang jing bo he	Boil in water for drinking, with ginger crushed and soaked in alcohol, add ginger to boil in water for drinking, mix with hot porridge	Treats wind-cold colds, expels wind	herb	cultivated	Aerial part, leaves	
140	LFYQ22689	Lamiaceae	*Ocimum basilicum* L	Bo he	Soak in alcohol for drinking, boil in water for drinking	Treats colds, coughs	herb	cultivated	Aerial part, leaves	
141	LFYQ23293	Lamiaceae	*Orthosiphon aristatus* (Blume) Miq	Miao xu cao	Boil in water for drinking	Treats sore throat, hepatitis, lumbago, kidney deficiency, nephritis, renal calculi; Has calculi-expelling, blood-lowering, and diuretic effects	herb	wild, cultivated	Aerial part	
142	LFYQ22483	Lamiaceae	*Perilla frutescens* (L.) Britton	Zi su	Boil in water for drinking	Treats wind-cold colds	herb	cultivated	Whole plant, aerial part	LC
143	LFYQ23629	Lamiaceae	*Prunella vulgaris* L	Xia ku cao	Boil in water for drinking	Treats breast hyperplasia, mastitis, and lung nodules, with heat-clearing, anti-inflammatory, and detoxifying effects	herb	wild	Whole plant	LC
144	LFYQ21003	Lamiaceae	*Vitex negundo* L	Wu zhi feng	Boil in water for washing	Treats colds, rheumatic bone pain, excessive sweating, used for postpartum wind syndrome, swelling-reduction, has anti-itch, damp-expelling, wind-expelling, and cold-expelling effects	shrub	wild	Branches	LC
145	LFYQ23055	Lardizabalaceae	*Sargentodoxa cuneata* (Oliv.) Rehd. & E. H. Wilson	Hong teng	Boil in water for drinking	Treats paralysis	liana	wild	Stem	
146	LFYQ23049	Lauraceae	*Cinnamomum cassia* (L.) D. Don	Rou gui, gui zhi	Boil in water for drinking	Treats weakness, body pain, foot pain; Has tonifying and blood-circulating effects	tree	cultivated	Branches	LC
147	LFYQ24313	Lauraceae	*Lindera aggregata* (Sims) Kosterm	Wu yao	Boil in water for drinking, boil in water for washing	Branches and leaves boiled in water for washing treat itching; Root boiled in water for drinking treats stomachache and bruises	tree	wild	Branches, roots	LC
148	LFYQ22213	Lauraceae	*Litsea cubeba* (Lour.) Pers	Mu jiang zi	Branches and leaves boiled in water for washing	Used for postpartum wind syndrome, promotes qi, and treats bruises	tree	wild	Branches	LC
149	LFYQ22238	Lauraceae	*Litsea glutinosa* (Lour.) C. B. Rob	Ye guo mu, sha tang guo	Boil in water for washing	Has wind-damp expelling effects; Treats neonatal skin diseases	tree	wild, cultivated	Branches	LC
150	LFYQ22011	Loranthaceae	*Macrosolen cochinchinensis* (Lour.) Tiegh	Huang pi mu ji sheng cha	Boil in water for washing	Has qi-promoting effects	shrub	wild	Whole plant	
151	LFYQ23575	Loranthaceae	*Taxillus chinensis* (DC.) Danser	Suan yang tao mu ji sheng, long yan mu ji sheng	Boil in water for drinking	Treats hepatitis, has qi-promoting effects	shrub	wild	Whole plant	
152	LFYQ22124	Lycopodiaceae	*Palhinhaea cernua* (L.) Vasc. & Franco	Song jin cao	With duck eggs in cooking, boil in water for drinking	Duck egg cooking treats cramps, boiling in water for drinking promotes bone-setting, clears heat, detoxifies, and treats ear inflammation, eczema, rheumatism, and itch	herb	wild	Whole plant	
153	LFYQ22164	Lygodiaceae	*Lygodium japonicum* (Thunb.) Sw	Niu dou xu	Boil in water for drinking	Treats renal calculi	herb	wild	Aerial part	
154	LFYQ22661	Malvaceae	*Abutilon indicum* (L.) Sweet	Ma ben mu, she mao mu, mo long zi	Boil in water for drinking; with crab in soup	Boil in water for drinking has diuresis, heat-clearing, and detoxifying effects; Treats tinnitus, renal calculi; With crab, it can treat pharyngitis	herb	wild, cultivated	Aerial part	
155	LYFQ21104	Malvaceae	*Ceiba speciosa* (A.St.-Hil., A.Juss. & Cambess.) Ravenna	Zuo deng feng	Boil in water for washing	Has wind-expelling effects	tree	cultivated	Branches	LC
156	LYFQ21155	Malvaceae	*Corchorus capsularis* L	Huang ma	Boil branches and leaves in water for washing, boil in water for drinking, use base stems and roots burned to ash and mix with water for drinking	Boiling in water for washing treats urticaria and beriberi, with anti-iching effects; Boiling in water for drinking treats kidney disease, hepatitis, and diarrhea; Ash from burned roots has hemostatic effects and treats hemorrhage	herb	wild	Aerial part	
157	LFYQ22249	Malvaceae	*Helicteres angustifolia* L	Po you ma	Use raw for external application, boil in water for hot compress, boil in water for drinking	Use raw for external application or hot compress to treat mosquito bites and mild pain; Has anti-itching and detoxifying effects, boiling in water for drinking treats internal cancers	shrub	wild	Branches	
158	LFYQ21104	Malvaceae	*Malvaviscus penduliflorus* DC	Hong hua, hong miao gao she	Boil in water for washing	Has blood-circulating effects	shrub	cultivated	Branches	
159	LFYQ23434	Malvaceae	*Sida rhombifolia* L	Bai shi ma tou	Boil in water for drinking, with sweetfish in cooking, root with honey in boiling water	Stems and leaves boiled in water for drinking to treat renal calculi and nephritis; Roots with honey in boiling water aid digestion; Sweetfish in cooking treats infantile m﻿alnutrition	herb	wild	Whole plant	
160	LFYQ23512	Malvaceae	*Urena lobata* L	Shi ma tou	Boil in water for washing	Has anti-itching effects	shrub	wild	Branches	LC
161	LFYQ22577	Marattiaceae	*Angiopteris fokiensis* Hieron	Ma ti	Boil in water for drinking	Has cough-relieving and cooling effects	herb	wild, cultivated	Rhizome	
162	LFYQ22296	Melanthiaceae	*Paris chinensis* Franch	Qi ye yi zhi hua	Boil in water for drinking	Treats chronic illness, gastrointestinal discomfort	herb	wild	Roots	
163	LYFQ21250	Melastomataceae	*Melastoma dodecandrum* Lour	Di nian	Boil in water for drinking, boil in water for washing	Boiling in water for drinking promotes blood circulation and treats diarrhea, boiling in water for washing treats bruises	shrub	wild	Whole plant	LC
164	LFYQ22178	Melastomataceae	*Melastoma sanguineum* Sims	Shan dou luo	Boil in water for washing	Treats body pain, foot pain, has hemostatic and injury-eliminating effects, used for postpartum wind syndrome, swelling reduction, pain relief, and hemostasis	shrub	wild	Branches	LC
165	LFYQ22179	Melastomataceae	*Melastoma malabathricum* L	Bao ya lang	Boil in water for washing	Has hemostatic, swelling-reducing, and pain-relieving effects, used for postpartum wind syndrome , swelling reduction, and pain relief; Treats itching, herpes, and bruises	shrub	wild	Branches	
166	LFYQ23541	Melastomataceae	*Osbeckia chinensis* L	Tian xiang lu, yang tian zhong	Boil in water for drinking, boil in water for washing, boil in water for hot compress	Boiling in water for drinking treats diphtheria, chronic bronchitis, pharyngitis; Boiling in water for washing and hot compress treats bruises, with swelling-reducing and pain-relieving effects	shrub	wild	Aerial part, whole plant	
167	LFYQ22427	Meliaceae	*Melia azedarach* L	Ku lian	Boil in water for washing	Has anti-itching effects	tree	cultivated	Bark	LC
168	LFYQ23221	Menispermaceae	*Cyclea barbata* Miers	Jin xian feng	Suck in the mouth, boil in water for drinking	Treats throat inflammation and sore throat; Has wind-expelling effects	liana	wild	Roots	
169	LFYQ22113	Menispermaceae	*Diploclisia affinis* (Oliv.) Diels	Cheng gou feng	Boil in water for drinking	Treats bruises and sprains	liana	cultivated	Vine leaves	
170	LFYQ22655	Menispermaceae	*Fibraurea recisa* Pierre	Huang lian teng, tu huang lian	Boil in water for drinking	Treats bloating, stomachache, has anti-inflammatory, heart-clearing, lung-moisturizing, cooling, and detoxifying effects, and relieves pain from bruises	liana	wild	Roots, stem	
171	LFYQ22541	Menispermaceae	*Stephania longa* Lour	Qian li teng	Boil in water for washing, boil in water for drinking	Boiling in water for washing treats scabies, pustules; Boiling in water for drinking treats sore throat, gastrointestinal inflammation, with heat-clearing, detoxifying, and pain-relieving effects	liana	wild	Aerial part	
172	LFYQ23552	Menispermaceae	*Stephania cephalantha* Hayata	Shan wu gui	Boil in water for drinking, crush for external application	Boiling in water for drinking treats snake bites, gastrointestinal diseases, has heat-clearing and detoxifying effects; Crush for external application treats bruises and scabies	liana	wild	Tuberous root	
173	LFYQ22455	Menispermaceae	*Tinospora crispa* (L.) Hook. f. & Thomson	Gen jing niang, yi cun wang	Boil in water for washing	Treats scabies, skin ulcers	liana	wild	Stem	
174	LFYQ23144	Menispermaceae	*Tinospora sinensis* (Lour.) Merr	Tian diao teng, wen jin teng, chun jing teng, kuan jin teng	With pig's feet or pig's tail in soup, boil in water for drinking, boil in water for washing	With pig's feet or pig's tail in soup, boiling in water for drinking treats sprains, strains, and cramps; Boiling in water for washing has tendon-soothing, swelling-reducing, and pain-relieving effects	liana	wild	Vine leaves	
175	LFYQ24141	Moraceae	*Broussonetia papyrifera* (L.) Vent	Wu ya tang	Boil in water for washing	Has anti-itching and anti-inflammatory effects	shrub	wild	Branches	LC
176	LFYQ24342	Moraceae	*Ficus triloba* Buch.-Ham. ex Voigt	Ye po feng	Boil in water for washing	Treats postpartum wind syndrome, with pain-relieving, blood-circulating, and wind-expelling effects	shrub	wild	Branches	LC
177	LFYQ23017	Moraceae	*Ficus simplicissima* Lour	Wu zhi mao tao	With bones in soup, soak in alcohol for drinking, boil in water for drinking	With bones in soup, treats kidney deficiency, expels dampness, tonifies weakness, drains pus, tonifies qi, strengthens the spleen, and has anti-inflammatory effects; Soaking in alcohol or boiling in water for drinking has antiviral, antibacterial, antitumor, wind-damp expelling, immune-boosting effects, and treats numbness in hands and feet	herb	wild	Roots	LC
178	LFYQ23373	Moraceae	*Ficus microcarpa* L. f	Rong mu xu	Boil in water for drinking	Treats lumbago, sprains, bruises	tree	cultivated	Aerial roots	LC
179	LFYQ22403	Moraceae	*Ficus pumila* L	Ba yao teng, liang fen zi	Boil in water for drinking	Treats cramps	shrub	wild, cultivated	Branches	
180	LFYQ22600	Moraceae	*Maclura cochinchinensis* (Lour.) Corner	Chuan po shi	Boil in water for drinking, with chicken or goose stomach membrane in cooking	Boiling in water for drinking treats lung diseases and calculi, has cancer-fighting effects; With chicken or goose stomach membrane, treats renal calculi and gallbladder calculi	shrub	wild	Roots, stem	
181	LFYQ22681	Moraceae	*Morus alba* L	Da sang ye	Boil in water for drinking	Treats cough, sore throat, conjunctivitis, has heat-clearing, lung-moisturizing, phlegm-expelling, and heat-toxicity-expelling effects	shrub	cultivated	Branches, leaves, bark, roots	LC
182	LFYQ23110	Myrtaceae	*Baeckea frutescens* L	Tie kia	Boil in water for washing, crush juice and apply, boil in water for drinking	Boiling the branches and leaves in water for washing itching and vaginal inflammation; Has antibacterial and anti-itch effects; Crushed tender leaves treats hornet stings; Boiling the root (after removing old skin) for drinking treats hepatitis	shrub	wild	Branches, young leaves, roots	LC
183	LYFQ21185	Myrtaceae	*Eucalyptus robusta* Sm	Da ye an	Boil in water for washing	Has antibacterial and anti-itch effects, treats skin diseases and B-type meningitis	tree	cultivated	Branches	NT
184	LFYQ24189	Nelumbonaceae	*Nelumbo nucifera* Gaertn	Lian peng	Boil in water for drinking	Has heat-clearing and detoxifying effects, expels fetal toxins in pregnant women	herb	cultivated	Flower receptacle	
185	LFYQ22067	Nephrolepidaceae	*Nephrolepis cordifolia (L.) C. Presl*	Shan huang pi	Eat raw, boil in water for drinking	Treats cough, chronic bronchitis	herb	wild, cultivated	Tuber	
186	LFYQ24143	Nyctaginaceae	*Mirabilis jalapa* L	Er huan cao	Boil in water for washing	Has anti-itching effects, treats cough	herb	cultivated	Aerial part	
187	LFYQ21014	Oleaceae	*Jasminum pentaneurum* Hand.-Mazz	Zhang ye mo li	Boil in water for drinking	Treats wind-heat syndrome, lack of bile	shrub	wild	Branches	
188	LFYQ22066	Onagraceae	*Ludwigia adscendens* (L.) Hara	Guo jiang long, guo tang she	Boil in water for washing, soak in rice water for application	Treats shingles	herb	wild	Whole plant	LC
189	LFYQ24128	Onagraceae	*Ludwigia perennis* L	Shui huang mu	Boil in water for washing	Reduces jaundice in children	herb	wild	Whole plant	LC
190	LFYQ22036	Orobanchaceae	*Striga asiatica* (L.) Kuntze	Du jiao jin	Boil in water for drinking	Treats infantile m﻿alnutrition	herb	wild	Whole plant	
191	LFYQ21004	Oxalidaceae	*Averrhoa carambola* L	Tian tao ye	Leaves with eggs in cooking, fruit pickled for consumption	Leaves with eggs in cooking water treat tooth pain and body pain; Pickled fruit treats gum swelling, oral ulcers, sore throat, and pharyngitis; Has stomach-strengthening, heat-clearing, and blood pressure-lowering effects	tree	cultivated	Branches, fruit	DD
192	LFYQ21064	Oxalidaceae	*Oxalis corniculata* L	Bai jiang cao, suan jin cao	Boil in water for washing	Treats bruises	herb	wild	Whole plant	
193	LFYQ22161	Pandanaceae	*Pandanus urophyllus* Hance	Shan bo luo, le gu	Boil in water for drinking, boil in water for soaking	Root boiled in water for soaking treats beriberi; Fruit boiled in water treats gastrointestinal discomfort, flatulence, hypertension, liver heat, hepatitis, painful urination, male urinary turbidity, calculi, hemorrhoids, urinary problems, hyperglycemia, bronchitis, and testicular underdevelopment in children, with heat-clearing, detoxifying, diuretic, and anti-inflammatory effects	shrub	wild, cultivated	Fruit, stem, roots	LC
194	LFYQ23508	Passifloraceae	*Passiflora cochinchinensis* Spreng	She yan teng	Crush for external application, boil in water for washing	Treats scabies, duodenal ulcers, shingles	liana	wild	Vine leaves	
195	LFYQ24035	Pentaphylacaceae	*Eurya nitida* Korth	Mi sui mu	Boil in water for washing	Treats skin diseases	shrub	wild	Branches	LC
196	LYFQ 21132	Phyllanthaceae	*Antidesma ghaesembilla* Gaertn	Mi sui mu	Boil in water for washing	Has heat-clearing and heat-toxicity-expelling effects	tree	wild	Branches	LC
197	LFYQ21099	Phyllanthaceae	*Bischofia javanica* Blume	Qiu feng	Boil in water for washing	Has wind-expelling and anti-weakness effects	tree	wild	Branches	LC
198	LFYQ23067	Phyllanthaceae	*Breynia fruticosa* (L.) Müll.Arg	Gui hua fu	Boil in water for drinking	Treats diarrhea; Treats gastrointestinal heat	shrub	wild	Branches	LC
199	LFYQ22547	Phyllanthaceae	*Glochidion zeylanicum* var. *tomentosum* (Dalzell) Trimen	Shui niu gan	Boil in water for washing	Treats excessive sweating	shrub	wild	Branches	
200	LFYQ21092	Phyllanthaceae	*Phyllanthus cochinchinensis* Spreng	Tie sao ba	Boil in water for washing	Has anti-itching effects	shrub	wild	Branches	
201	LYFQ21174	Phyllanthaceae	*Phyllanthus emblica* L	You gan zi	Fruit eaten raw, skin boiled with pig's feet in soup, branches and leaves boiled in water for washing	Fruit eaten raw moistens the lungs and relieves cough; Bark boiled with pig's feet in soup treats diabetes; Branches and leaves boiled in water for washing treat infantile m﻿alnutrition	tree	wild	Bark, fruit, branches	LC
202	LFYQ22536	Phyllanthaceae	*Phyllanthus glaucus* Wall. ex Müll.Arg		Boil in water for washing	Treats children's loss of appetite, infantile m﻿alnutrition	shrub	wild	Branches	
203	LFYQ21030	Phyllanthaceae	*Phyllanthus urinaria* L	Bei bei gan	Boil in water for drinking	Treats kidney inflammation, has swelling-reducing effects	herb	wild	Whole plant	
204	LFYQ24062	Phyllanthaceae	*Flueggea gracilis* (Merr.) Petra Hoffm	Hong rong dan, hong pi xiong dan	Boil in water for drinking	Treats sore throat, has liver-cooling, lung-cooling, and heat-reducing effects	tree	wild	Roots	LC
205	LFYQ23169	Pinaceae	*Pinus massoniana* Lamb	Chong mu, chong jie	Boil in water for drinking	Boil stem inner endodermis for drinking to treat bruises, diabetes; Root boiled in water for drinking treats rheumatic bone pain; Tender branches boiled in water for drinking treat iron injury, has hemostatic effects; Joints boiled in water for drinking treat bruises and joint pain	tree	cultivated	Stem endodermis, roots, young stems, nodes	LC
206	LFYQ23245	Piperaceae	*Piper hongkongense* C. DC	Mao le	Boil in water for drinking	Treats bruises and stomachache	liana	wild	Whole plant	
207	LFYQ24028	Piperaceae	*Piper hancei* Maxim	Shang shan hu	Boil in water for drinking, boil in water for washing, soak in water for drinking, soak in alcohol for rubbing	Boiling in water for washing treats wind-paralysis, reduces swelling; Boiling in water for drinking and soaking in water for drinking treats colds, gastrointestinal inflammation; Soaking in alcohol for rubbing treats bruises, bone hyperplasia	herb	wild	Whole plant	
208	LFYQ22039	Plantaginaceae	*Plantago asiatica* L	Che qian cao	Boil in water for drinking	Treats painful urination, calculi, and urinary pain, has diuretic, hemostatic, and heat-clearing effects	herb	wild	Whole plant	
209	LFYQ23511	Plantaginaceae	*Scoparia dulcis* L	Bing tang cao, fei zi cao	Boil in water for washing, boil in water for drinking	Boiling in water for drinking clears heat and detoxifies; Boiling in water for washing expels wind and treats skin diseases and prickly heat	herb	wild	Whole plant	
210	LFYQ22060	Plumbaginaceae	*Plumbago zeylanica* L	Bai hua dan	Boil in water for washing, boil in water for hot compress, with bones in soup	Boiling in water for washing and hot compress treats joint hyperplasia; With bones in soup treats infantile m﻿alnutrition and drooling	shrub	wild	Branches	
211	LFYQ21024	Poaceae	*Axonopus compressus* (Sw.) P. Beauv	Hai di chang, ying gen cao	Eat leaves raw, boil the whole plant in water for drinking	Eating leaves raw treats stomachache and indigestion; Boiling the whole plant in water for drinking has heat-clearing and detoxifying effects	herb	wild	Leaves, whole plant	LC
212	LFYQ22535	Poaceae	*Bambusa chungii* McClure	Dan zhu ye	Boil in water for washing	Has heat-clearing and detoxifying effects	herb	wild	Branches	
213	LYFQ21278	Poaceae	*Cymbopogon citratus* (DC.) Stapf	Xiang mao	Boil in water for hot compress, boil in water for washing	Boiling in water for washing treats foot pain, colds, and coughs; Hot compress with boiled water treats bruises	herb	wild	Aerial part	
214	LFYQ23080	Poaceae	*Imperata cylindrica* (L.) Raeusch	Bai mao gen	Boil in water for drinking, boil in water for washing	Root boiled in water for drinking treats heat-clearing, detoxifying, liver-cooling, blood-cooling, hemostatic, and cough-relieving effects; Treats cold, fever, and gastrointestinal heat; Aerial parts boiled in water for washing expels dampness and relieves itching	herb	wild	Roots, aerial part	
215	LYFQ21166	Poaceae	*Lophatherum gracile* Brongn	Shan ji mi	Boil in water for drinking, boil in water for washing	Boiling in water for drinking has heat-clearing, detoxifying, phlegm-expelling, and nourishing yin effects; Treats high fever; Boiling in water for washing has heat-clearing and detoxifying effects	herb	wild	Whole plant	
216	LFYQ22330	Polygalaceae	*Polygala fallax* Hemsl	Huang hua dao shui lian	Boil in water for drinking	Treats sore throat	shrub	wild	Roots	LC
217	LFYQ22739	Polygalaceae	*Polygala chinensis* L	Jin bu huan, zi bei jin niu, nang yao	Soak in water for drinking, boil in water for drinking	Treats neurasthenia, insomnia, kidney inflammation, children's infantile m﻿alnutrition, with anti-inflammatory, liver-protecting, stomach-strengthening, and kidney-function-regulating effects	herb	wild	Whole plant	
218	LFYQ23585	Polygalaceae	*Securidaca inappendiculata* Hassk	Xue pi teng	Boil in water for drinking, boil in water for washing	Boiling in water for washing treats body itching; Boiling in water for drinking treats body pain, foot pain	shrub	wild	Branches	
219	LFYQ22150	Polygonaceae	*Fagopyrum acutatum* Mansf. ex K.Hammer	Shan jiu cai	Boil in water for drinking	Has heat-clearing and detoxifying effects	herb	wild	Aerial part	
220	LFYQ21012	Polygonaceae	*Muehlenbeckia platyclada* (F. Muell. ex Hook.) Meisn	Fei tian wu gong	Sun-dried and soaked in high alcohol for external application, crush stems and leaves for external application, boil in water for drinking	Crush stems and leaves for external application to treat tooth pain; Sun-dried root soaked in alcohol for external application hemostasis and treats numbness; Leaves boiled in water for drinking treats snake venom	shrub	cultivated	Roots, branches, leaves	
221	LFYQ22693	Polygonaceae	*Persicaria hydropiper* (L.) Spach	La liao, tie liao	Boil in water for washing, boil in water for hot compress, boil in water for drinking	Treat bruises, has anti-itching, anti-inflammatory, and anti-bacterial effects	herb	wild	Whole plant	LC
222	LYFQ21284	Polygonaceae	*Persicaria palmata* (Dunn) Yonek. & H. Ohashi	E zhang feng	Boil in water for washing	Treats cracked hands and feet, calluses	herb	wild	Whole plant	
223	LFYQ22184	Polygonaceae	*Persicaria chinensis* (L.) H. Gross	Huo zhi tan cha	Boil in water for washing, boil in water for drinking	Boiling in water for drinking clears heat and detoxifies; Boiling in water for washing treats uterine hemorrhage , has hemostatic and anti-itching effects	herb	wild	Aerial part	
224	LFYQ22122	Polygonaceae	*Polygonum perfoliatum* L	Gang ban gui	Boil in water for washing	Has anti-itching effects	herb	wild	Whole plant	
225	LFYQ22749	Polygonaceae	*Reynoutria multiflora* (Thunb.) Moldenke	Ye jiao teng	Boil in water for drinking	Treats wind-paralysis	herb	wild	Tuberous root	
226	LFYQ23156	Polygonaceae	*Polygonum plebeium* R. Br	Wu ying yi	Boil in water for drinking	Treats lumbar disc herniation, wind-paralysis, has tendon-soothing and circulation-promoting effects	herb	wild	Whole plant	LC
227	LFYQ23592	Polygonaceae	*Reynoutria japonica* Houtt	Guan jiao	Boil in water for washing, boil in water for drinking	Boiling in water for drinking treats hepatitis, has detoxifying and damp-expelling effects; Boiling in water for washing treats bruises, has tendon-soothing, anti-inflammatory effects, and expels dampness	herb	wild	Rhizome	
228	LFYQ22573	Polypodiaceae	*Drynaria roosii* Nakaike	Gu sui bu	Remove hair and cook with bones in soup	Removes hair, stews with bones to treat lumbar disc herniation, lumbar hyperplasia, lumbago, rubella , eczema	herb	wild	Rhizome, leaves	
229	LFYQ22433	Pontederiaceae	*Pontederia crassipes* Mart	Fu piao	Boil in water for washing	Has anti-itch effects	herb	wild	Whole plant	
230	LFYQ21063	Portulacaceae	*Portulaca oleracea* L	Ma shi han	With eggs in cooking	Has stomach-strengthening, digestion-aiding, and constipation-relieving effects	herb	wild	Whole plant	LC
231	LFYQ22611	Primulaceae	*Ardisia crenata* Sims	Da luo san	Boil in water for washing, heat for external application	Boiling in water for washing can expel injury accumulation, treat bruises; with anti-inflammatory and pain-relieving effects; Heat application treats bruises and sprains	shrub	wild	Whole plant	
232	LFYQ23343	Primulaceae	*Ardisia gigantifolia* Stapf	Zou ma feng	Boil in water for washing	Has wind-expelling effects	shrub	wild	Branches	
233	LFYQ22137	Primulaceae	*Ardisia japonica* (Thunb.) Blume	Bu chu lin, ai di cha	With pig's lung in soup; Boil in water for drinking	With pig's lung, it treats cough; Boiling in water for drinking treats bruises and sprains	shrub	wild	Whole plant	
234	LFYQ22077	Primulaceae	*Ardisia lindleyana* D. Dietr	Shan xue dan	Boil in water for drinking	Treats bruises and sprains, gastrointestinal damp-heat; Has pain-relieving effects	shrub	wild	Whole plant	
235	LFYQ23207	Primulaceae	*Ardisia villosa* Roxb	Ai jiao luo san, a mao guo, xiao luo san	Boil in water for external application, soak in alcohol for rubbing, boil in water for drinking	Boiling water for drinking treats throat diseases; for washing and soaking in alcohol for rubbing treats joint pain, bone hyperplasia, bruises, sprains; with swelling-reducing and pain-relieving effects	shrub	wild	Whole plant	
236	LFYQ24269	Primulaceae	*Embelia laeta* (L.) Mez	Bao zi guo	Boil branches and leaves in water for washing, roots boiled in water for drinking	Boiling the branches and leaves in water for washing treats eczema, sores; Boiling the branches and leaves in water for drinking has digestive effects; Boiling the root for drinking has anti-inflammatory effects	shrub	wild	Branches, roots	
237	LYFQ21129	Primulaceae	*Maesa japonica* (Thunb.) Moritzi ex Zoll	Du jing shan	Boil in water for washing	Has wind-expelling, diuretic, hemostatic, and swelling-reducing effects	shrub	wild	Branches	
238	LFYQ22345	Pteridaceae	*Pteris semipinnata* L	Wu jiao qing	Boil in water for washing	Has heat-clearing and detoxifying effects	herb	wild	Whole plant	
239	LFYQ23357	Rhamnaceae	*Berchemia lineata* DC	Miao shi guo	Boil in water for washing, boil in water for drinking	Boiling in water for washing has anti-itch effects; Boiling in water for drinking treats chronic bronchitis	shrub	wild	Branches	
240	LFYQ24067	Rhamnaceae	*Frangula crenata* (Siebold & Zucc.) Miq	Shan la liao, hu li jing	Boil in water for washing	Has anti-inflammatory, anti-bacterial, anti-itching, detoxifying, and wind-damp expelling effects	shrub	wild	Roots, stem	LC
241	LFYQ22146	Rosaceae	*Agrimonia pilosa* Ledeb	Xian he cao	Boil in water for drinking	Treats dysmenorrhea, excessive vaginal discharge, uterine hemorrhage, irregular menstruation	herb	wild, cultivated	Whole plant	
242	LFYQ22691	Rosaceae	*Eriobotrya japonica* (Thunb.) Lindl	Pi ba	Boil in water for drinking	Has lung-cooling effects, treats cough	tree	cultivated	Leaves	
243	LFYQ21102	Rosaceae	*Prunus persica* (L.) Batsch	Tao ye	Boil in water for washing, newborns suspended at the door	Hanging at the door prevents evil spirits; Boiling in water for washing relieves itching	tree	cultivated	Branches	
244	LFYQ22153	Rosaceae	*Rosa laevigata* Michx	Jin ying zi	Soak in alcohol for drinking, with bones in soup	With bones in soup, has anti-inflammatory, blood-toning, and kidney-toning effects, treats stomach dampness; Soaking in alcohol for drinking tonifies the kidneys, treats frequent urination, urgency urination, abnormal leucorrhea, low sperm count, lumbago	shrub	wild	Fruit, roots	
245	LFYQ22185	Rosaceae	*Rubus cochinchinensis* Tratt	Ci wu jia	Boil in water for drinking	Has kidney-toning and liver-toning effects	shrub	wild	Branches	
246	LFYQ22440	Rubiaceae	*Adina pilulifera* (Lam.) Franch. ex Drake	Shui yang mei	Boil in water for washing	Treats hemorrhoids, itching, rheumatism, eczema, heat-toxicity; Has heat-clearing and detoxifying effects	shrub	wild	Branches	LC
247	LFYQ22272	Rubiaceae	*Aidia canthioides* (Champ. ex Benth.) Masam	Yan ji wei	Boil in water for washing; Tender leaves with hot glutinous rice for poultice	Boiled leaves for washing treat postpartum wind and bruises; warm poultice of tender leaves with glutinous rice relieves gout and edema; bark decoction wash helps rheumatic bone pain	tree	wild	Branches, bark	LC
248	LFYQ23475	Rubiaceae	*Canthium horridum* Blume	Zhu du mu	Crush root for poultice; Root boiled in water for drinking; Fruit with meat in cooking	Crush root for poultice to treat snakebites; Boil root for drinking to treat calculi and stomach diseases; Boil fruit with meat to treat malnutrition	shrub	Wild	Roots, fruit	
249	LFYQ23605	Rubiaceae	*Gardenia jasminoides* J. Ellis	Zhi zi	Boil in water for washing, boil in water for drinking	Treats jaundice, has heat-clearing and detoxifying effects	shrub	wild, purchased	Branches	LC
250	LFYQ22040	Rubiaceae	*Hedyotis caudatifolia* Merr. & F. P. Metcalf	Long gou gan	Boil in water for drinking	Treats liver and gallbladder heat, eye heat, has heat-clearing and detoxifying effects	herb	wild	Aerial part	
251	LYFQ21028	Rubiaceae	*Oldenlandia corymbosa* L	Liao ge she, she she cao	Boil in water for drinking, add egg in cooking	Treats snake venom, appendicitis, has diuretic effects	herb	wild	Whole plant, aerial part	
252	LFYQ22015	Rubiaceae	*Mussaenda pubescens* Dryand	Xi liang teng	Branches and leaves boiled in water for washing, roots boiled in water for drinking	Roots and branches and leaves boiled in water for drinking have heat-clearing and detoxifying effects; Branches and leaves boiled in water for washing to treat itch	shrub	wild	Branches, roots	
253	LFYQ24140	Rubiaceae	*Paederia foetida* L	Ji shi teng	Boil in water for washing, boil in water for drinking	Expels wind, treats colds	liana	wild, cultivated	Aerial part	
254	LFYQ21134	Rubiaceae	*Psychotria asiatica* L	Lu gu qing, dao gu qing	Boil in water for drinking, soak in water for drinking	Stems, leaves, and roots boiled in water for soaking treats bruises and injuries; Tender leaves soaked in water for drinking treats diarrhea	shrub	wild	Branches, roots, young leaves	LC
255	LFYQ23497	Rubiaceae	*Scleromitrion diffusum* (Willd.) R. J. Wang	She she cao	Boil in water for drinking	Treats hepatitis, cirrhosis, snakebite	herb	wild	Whole plant	
256	LFYQ24139	Rubiaceae	*Spermacoce alata* Aubl	Ri ben cao	Boil in water for washing	Has anti-itching effects	herb	wild	Whole plant	
257	LFYQ22696	Rubiaceae	*Tarenna mollissima* (Hook. & Arn.) B. L. Rob	Wu mu	Boil in water for drinking	Has blood-moving and pain-relieving effects	tree	wild	Stem	LC
258	LFYQ21114	Rubiaceae	*Uncaria macrophylla* Wall	Gou teng	Boil in water for washing	Has wind-expelling effects	liana	wild	Vine leaves, hooks	
259	LFYQ22097	Rubiaceae	*Uncaria rhynchophylloides* F. C. How	Gou teng	Boil in water for drinking, boil in water for washing	Boiling in water for drinking treats dysmenorrhea, boiling in water for washing expels wind	liana	wild	Roots, vine leaves, hooks	
260	LFYQ23250	Rutaceae	*Atalantia buxifolia* (Poir.) Oliv	Ku le ding, gui gan le	Boil in water for drinking	Treats hepatitis, cirrhosis, renal calculi	shrub	wild	Branches	LC
261	LFYQ21107	Rutaceae	*Citrus reticulata* Blanco	Gan mu	Boil in water for washing	Has effects of promoting qi and blood circulation	tree	cultivated	Branches	
262	LFYQ21118	Rutaceae	*Clausena excavata* N. L. Burman	Shan huang pi	Boil in water for washing	Treats children’s night crying, has jaundice-removing effects	shrub	wild	Branches	LC
263	LFYQ22441	Rutaceae	*Melicope pteleifolia* (Champ. ex Benth.) Hartley	San cha ku	Boil in water for drinking, boil in water for washing	Boiling in water for drinking clears heat, detoxifies, treats colds, wind sha , flu, and encephalitis; Boiling in water for washing clears heat, detoxifies, expels wind, relieves itching, and treats bruises, used for postpartum wind syndrome, swelling reduction	tree	wild	Branches	LC
264	LFYQ23276	Rutaceae	*Micromelum minutum* (G.Forst.) Wight & Arn	Bai mu	Boil in water for drinking	Expels injury accumulation	shrub	wild	Stem	LC
265	LFYQ21106	Rutaceae	*Murraya exotica* L	Ba bai li	Boil in water for washing	Treats bruises, has injury-eliminating, swelling-reducing, and pain-relieving effects	tree	cultivated	Branches	
266	LFYQ22637	Rutaceae	*Phellodendron chinense* var. *glabriusculum* C. K. Schneid	Huang bo	Boil in water for drinking, boil in water for washing	Boiling in water for washing clears heat, expels dampness, and treats fungal skin diseases; Boiling in water for drinking treats liver and gallbladder damp heat, hepatitis	tree	wild, purchased	Stem	
267	LFYQ23573	Rutaceae	*Toddalia asiatica* (L.) Lam	Fei long zhang xue	Boil in water for washing	Treats bruises, has blood-dispersing effects	liana	wild	Stem	
268	LFYQ22111	Rutaceae	*Zanthoxylum nitidum* DC	Liang mian zhen	Boil in water for drinking, boil in water for hot compress, boil in water for washing, chew for oral use	Boiling in water for drinking treats bruises, tooth pain, stomachache, has pain-relieving and anti-inflammatory effects; Boiling in water for hot compress or boiling in water for washing treats foot swelling, bruises, has swelling-reducing and pain-relieving effects; Boiling in water for drinking or chewing treats tooth pain	liana	wild	Roots, vine leaves	LC
269	LFYQ24296	Santalaceae	*Viscum liquidambaricola* Hayata	San cha feng ji sheng	Boil in water for drinking	Treats rheumatic bone pain, gout	shrub	wild	Whole plant	
270	LFYQ24144	Sapindaceae	*Dimocarpus longan* Lour	Long yan mu	Boil in water for washing, eat raw, dry in the sun, boil in soup for drinking	Boiling the branches and leaves for washing in water treats itch; Used for postpartum wind syndrome; eating fruits or boiling the fruits in soup has tonifying effects	tree	cultivated	Branches, fruit	DD
271	LFYQ22493	Sapindaceae	*Sapindus saponaria* L	Xi shou guo	Boil in water for drinking, boil in water for washing	Boiling in water for drinking treats stomachache, sore throat, chronic pharyngitis, has anti-diarrheal effects; Boiling in water for washing has anti-inflammatory effects	tree	wild, purchased	Fruit	LC
272	LFYQ22569	Sapotaceae	*Manilkara zapota* (L.) van Royen	Xian ren guo	Eat raw, boil in water for drinking	Has blood-toning effects	tree	cultivated	Fruit	LC
273	LFYQ23035	Saururaceae	*Houttuynia cordata* Thunb	Yu xing cao	Boil in water for drinking, chew and apply	Boiling in water for drinking clears the heart and moistens the lungs, treats cough and phlegm; Chew and apply the remainder to wounds to relieve snake venom	herb	cultivated	Whole plant	
274	LFYQ23596	Schisandraceae	*Kadsura coccinea* (Lem.) A. C. Sm	Hei lao hu, xi yang chun jin teng, fan tuan guo	Boil in water for drinking, add pig's feet in soup	Pig's feet soup treats diabetes, excessive dampness; Branches and leaves boiled in water treat gastritis, stomach qi, with kidney-toning, pain-relieving, stomach-strengthening, and bone-strengthening effects; Root boiled in water for drinking treats pain and expels wind	liana	wild, cultivated	Vine leaves, roots	
275	LFYQ23048	Schisandraceae	*Kadsura heteroclita* (Roxb.) Craib	Hai feng teng	Boil in water for drinking	Has wind-expelling effects	liana	wild	Stem	
276	LFYQ24063	Schisandraceae	*Kadsura longipedunculata* Finet & Gagnep	Xiao zuan	Boil in water for drinking, add pig's feet in soup, boiling for hot compress	Boiling in water for drinking treats stomachache, viral skin diseases, and toxic sores; Has heat-clearing and detoxifying effects; Pig's feet soup treats rheumatism, bruises; Boiling for hot compress treats bruises with anti-inflammatory effects	liana	wild	Roots, vine leaves	
277	LFYQ22119	Selaginellaceae	*Selaginella moellendorffii* Hieron	Tai rong	Boil in water for washing	Treats chickenpox, neonatal fetal toxins	herb	wild	Whole plant	
278	LFYQ22167	Selaginellaceae	*Selaginella pulvinata* (Hook. & Grev.) Maxim	Tai du cao, huan hun cao	Boil in water for washing	Treats neonatal fetal toxins	herb	wild	Whole plant	
279	LFYQ22551	Selaginellaceae	*Selaginella rolandi-principis* Alston	Shi bi cao	With tofu and rock candy in cooking, boil in water for drinking	Treats hepatitis, liver heat	herb	wild	Whole plant	
280	LFYQ23231	Smilacaceae	*Smilax china L*	Jin gang le, ma ge le	With bones in soup, soak in alcohol for drinking	With bones in soup, tonifies blood; Soaking in alcohol for drinking clears heat, expels wind-damp, and soothes tendons and promotes circulation	shrub	wild	Rhizome	
281	LFYQ22118	Smilacaceae	*Smilax glabra* Roxb	Tu fu ling	Boil in water for drinking, with pig's tail in soup	Boiling in water for drinking expels dampness, tonifies, treats jaundice; With pig's tail in soup, expels dampness, opens joints, treats syphilis, lumbago	shrub	wild	Rhizome	
282	LFYQ22149	Smilacaceae	*Smilax riparia* A. DC	Qian jin ba	Boil in water for drinking	Root boiled in water for drinking promotes bone-setting, soothes tendons, relieves cough and phlegm; Treats lumbago; Branches and leaves boiled in water for drinking can treat diabetes	liana	wild	Roots, vine leaves	
283	LFYQ22322	Solanaceae	*Physalis angulata* L	Da bo cao	Boil in water for washing, boil in water for drinking	Boiling in water for washing treats chickenpox; Boiling in water for drinking treats flatulence, wind-stroke, and sore throat	herb	wild	Whole plant	LC
284	LFYQ21029	Solanaceae	*Solanum americanum* Mill	Bai hua cai	Boil in water for drinking	Has heat-clearing and detoxifying effects, relieves cough	herb	wild	Young leaves	
285	LFYQ24349	Solanaceae	*Solanum procumbens* Lour	Er huan tao gen	Add sweetfish in cooking	Treats food accumulation, infantile malnutrition	shrub	wild	Vine leaves	
286	LFYQ22270	Thelypteridaceae	*Thelypteris simplex* (Hook.) K.Iwats	Cao xie qing	Boil in water for drinking	Treats diarrhea	herb	wild	Whole plant	
287	LFYQ24064	Thymelaeaceae	*Wikstroemia indica* (L.) C. A. Mey	Ji zi ma	Boil in water for drinking, boil in water for washing	Treats Sha, scabies	shrub	wild	Branches	
288	LFYQ22522	Urticaceae	*Boehmeria nivea* (L.) Gaudich	Zhu ma	Boil in water for washing	Treats chickenpox and measles; Has anti-itching effects	shrub	wild	Branches	
289	LYFQ21181	Verbenaceae	*Lantana camara* L	Wu se hua	Boil in water for washing	Has wind-expelling and anti-itching effects	shrub	wild	Branches	
290	LFYQ21077	Verbenaceae	*Verbena officinalis* L	Ma bian cao	Boil in water for drinking	Has heat-clearing and detoxifying effects, treats gastrointestinal diseases and calculi	herb	wild	Whole plant	
291	LFYQ22510	Viburnaceae	*Sambucus javanica* Reinw. ex Blume	Zou ma feng, zou ma jian	Boil in water for washing	Has wind-expelling and detoxifying effects	herb	wild	Aerial part	LC
292	LFYQ24087	Viburnaceae	*Viburnum odoratissimum* Ker Gawl	Lei pian mu	Boil in water for washing	Has wind-expelling and wind-damp dispelling effects, treats scabies and breast carbuncle	tree	wild	Branches	LC
293	LFYQ22686	Violaceae	*Viola japonica* Langsd. ex Ging	Di ding	Crush for external application, boil in water for drinking	Crush for external application treats boils and breast carbuncle; Boil in water for drinking treats purulent osteomyelitis, jaundice, red-eye disease, with heat-clearing and detoxifying effects	herb	wild	Whole plant	
294	LFYQ23540	Vitaceae	*Cissus hexangularis* Thorel ex Planch	Kuan jin teng, liu fang teng, shen jin teng, chun jin teng	Boil in water for washing, boil in water for drinking	Has effects of soothing tendons and promoting circulation	liana	wild, cultivated	Vine leaves	
295	LFYQ23537	Vitaceae	*Cissus pteroclada* Hayata	Chou jin teng, kuan jin teng, shen jin teng, si fang teng	With bones in soup, with pig's trotters in cooking, crush and apply	With bones in soup to soothe tendons and promote circulation; With pig's trotters or crushed leaves to treat cramps	liana	wild, cultivated	Vine leaves	
296	LFYQ24160	Vitaceae	*Cissus repens* Lam	Bai fen teng	Boil in water for washing	Has soothing tendons and promoting circulation effects	liana	wild	Vine leaves	
297	LFYQ24164	Vitaceae	*Tetrastigma planicaule* (Hook. f.) Gagnep	Bian gu feng	Boil in water for drinking	Treats numbness in hands and feet	liana	wild	Stem	
298	LFYQ24094	Zingiberaceae	*Alpinia officinarum* Hance	Shan jiang	Boil in water for drinking	Has phlegm-expelling, heat-clearing, and qi-promoting effects	herb	cultivated	Roots	
299	LFYQ24154	Zingiberaceae	*Alpinia zerumbet* (Pers.) B. L. Burtt & R. M. Sm	Sha ren	Boil in water for drinking	Treats diarrhea, vomiting; Has wind-expelling effects	herb	wild, cultivated	Aerial part	DD
300	LFYQ21142	Zingiberaceae	*Curcuma longa* L	Huang jiang	With meat stir-fried for consumption, boil in water for hot compress, boil in water for drinking, boil in water for washing	Stir-fried with meat expel wind, hot compress with boiled water to relieve pain and treat bruises, boiling in water for drinking treats stomachache, boiling in water for washing treats jaundice	herb	cultivated	Rhizome	DD
301	LFYQ24158	Zingiberaceae	*Curcuma phaeocaulis* Valeton	Wu xin jiang	Boil in water for drinking	Has anti-inflammatory, tissue-regenerating, and pain-relieving effects; Treats women’s lumbago and gout	herb	cultivated	Rhizome	DD
302	LFYQ22032	Zingiberaceae	*Curcuma aromatica* Salisb	Feng jiang, ma lou jiang	With meat stir-fried for consumption, boil in water for drinking, add chicken or duck for stir-frying	Stir-fried with meat to expel wind, boiling in water for drinking treats rheumatic bone pain; Stir-frying with chicken or duck promotes qi and treats lumbar hyperplasia, lumbago, and joint hyperplasia	herb	cultivated	Rhizome	
303	LFYQ24182	Zingiberaceae	*Kaempferia rotunda* L	Tian qi	Boil in water for drinking	Has menstrual-regulating and hemostatic effects	herb	cultivated	Rhizome	
304	LFYQ22031	Zingiberaceae	*Zingiber zerumbet* (L.) Sm	Sheng jiang	Boil in water for drinking, with chicken, pig's stomach, and other meats cooked in wine, boil in water with wine for washing	Boiling in water for drinking expels wind, treats colds; With chicken, pig's stomach, and other meats cooked in wine for drinking, used for postpartum wind syndrome and pain relief,boiling in water with wine for washing to use for post-partum recovery	herb	cultivated	Rhizome	DD
305	LFYQ24159	Zingiberaceae	*Zingiber zerumbet* (L.) Roscoe ex Sm	Fu shou gun	Boil in water for washing	Has wind-expelling effects, treats rheumatic bone pain	herb	cultivated	Rhizome	

### Life forms and parts of plants used

**Fig. 3:**
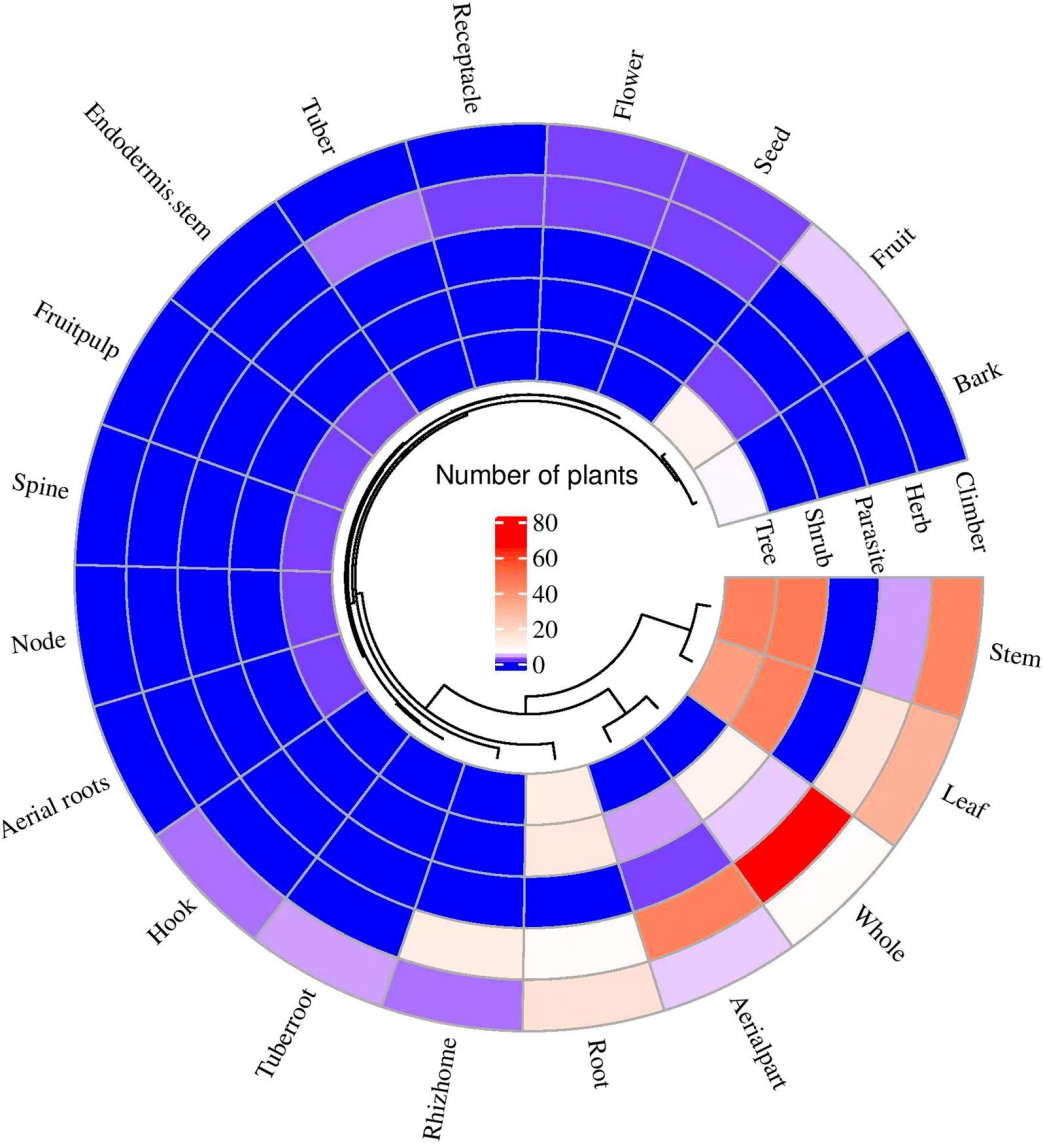
Circular plot showing the relationship between life forms and parts used

The majority of medicinal plants sold at markets in the Hakka communities of Southeastern Guangxi Province of China are herbs, comprising 130 species (42.62%), followed by shrubs with 82 species (26.89%), climbers with 47 species (15.41%), and trees with 46 species (15.08%). Regarding the plant parts utilized, leaves are the most frequently used, accounting for 122 species (32.36%), followed by whole plants for 84 species (22.28%), roots for 62 species (16.45%), aerial parts for 52 species (13.79%), and stems for 28 species (7.43%). Whole plants are predominantly used among herbaceous species, representing 70 species (53.85%) out of the 130 herb species recorded (Fig. 3).

### Plants used in the treatment of diseases and ailments

**Fig. 4:**
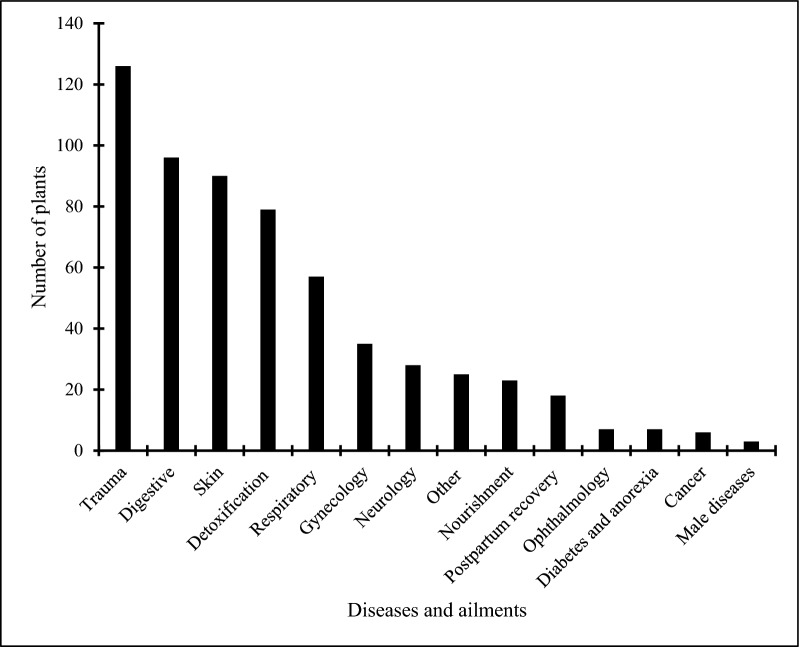
Number of plants reported to treat diseases and ailments

The recorded medicinal plants (305 species) exhibit 63 therapeutic effects and are utilized to treat 117 disease ailments across 14 categories. Of them, most of the plants were used in the treatment of physical trauma (Trauma) 126 species (41.31%), followed by digestive diseases 96 species (31.47%), skin diseases 90 species (29.51%), detoxification 79 species (25.90%), respiratory 57 species (18.69), neurology 28 species (9.18%), other 25 species (8.19%), nourishment 23 species (7.54%), postpartum recovery 18 species (5.9%), Ophthalmology seven species (2.29%), diabetes and anorexia seven species (2.29%), cancer six species (1.97%) and male disease three species (0.98%) (Fig. 4).

### Quantitative indices

**Fig. 5:**
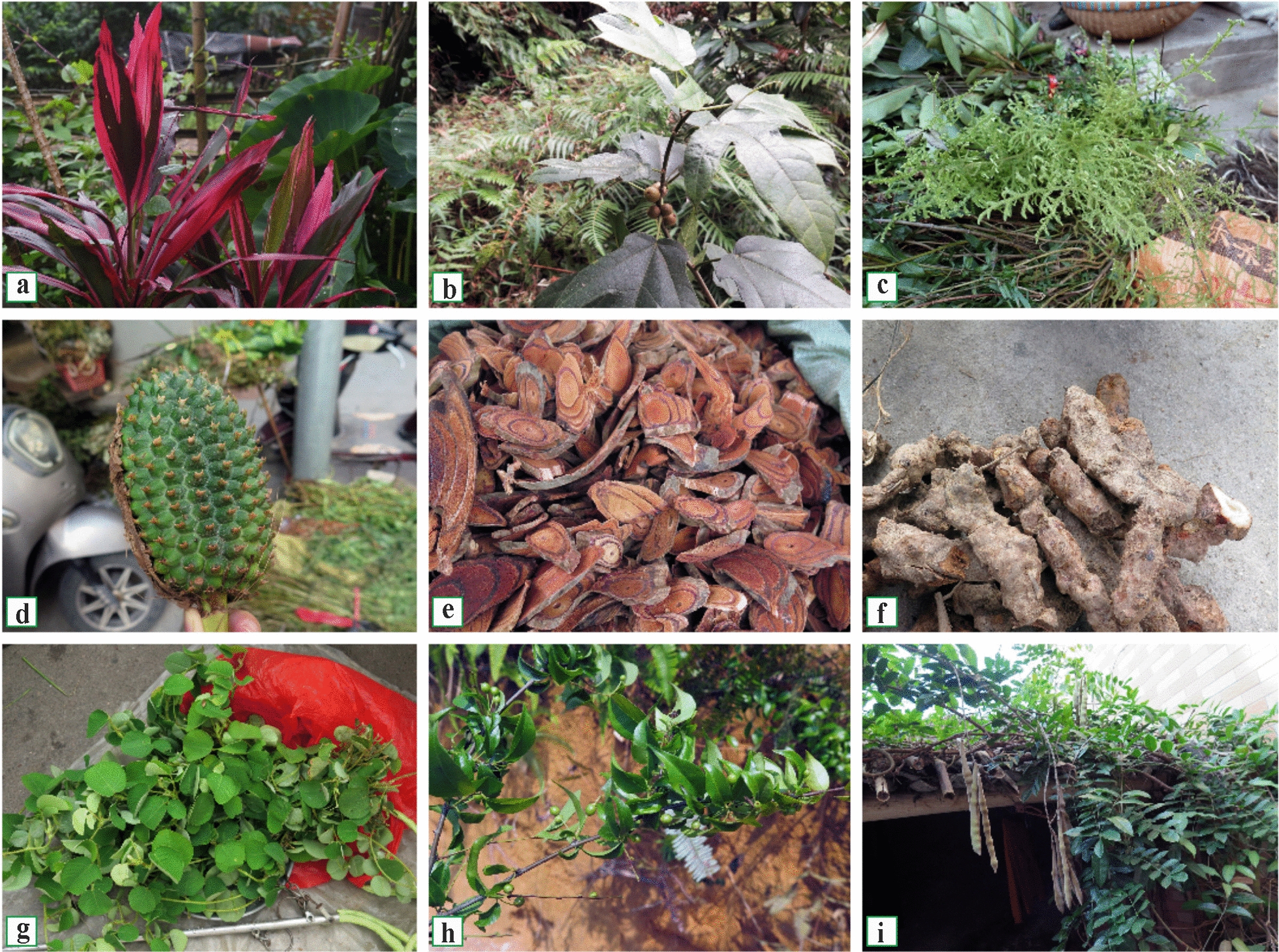
Some medicinal species with high RFC and RI values (**a ***Cordyline fruticosa* (L.) A. Chev.; **b** *Ficus simplicissima* Lour.; **c** *Palhinhaea cernua* (L.) Vasc. & Franco; **d** *Pandanus urophyllus* Hanc; **e** *Spatholobus suberectus* Dunn; **f** *Smilax glabra* Roxb.; **g** *Grona styracifolia* (Osbeck) H. Ohashi & K. Ohashi; **h** *Ilex asprella* Champ. Ex Benth.; **i** *Nanhaia speciosa* (Champ. ex Benth.) J. Compton & Schrire)

A total of 28 medicinal plants sold in traditional markets of the Southeastern Guangxi Hakka region exhibit high citation frequencies (RFC ≥ 0.5), with *Smilax glabra*, *Ilex asprella*, *Ficus simplicissima*, *Grona styracifolia*, and *Spatholobus suberectus* demonstrating notably elevated citation rates (some images are shown in Fig. 5). Among these 28 highly cited species, the predominant therapeutic categories include wound healing (15 species), heat-clearing and detoxification (10 species), digestive remedies (10 species), respiratory treatments (10 species), and postpartum wind-expulsion (8 species).

We documented 23 medicinal plants with relative importance (RI) values ≥ 1.25, among which *Cordyline fruticosa*, *Ficus simplicissima*, *Spatholobus suberectus*, *Pandanus urophyllus*, and *Palhinhaea cernua* exhibit notably high RI values (Fig. 5). These species are prevalent in the region and are widely recognized by the local population.

### Comparative analysis

**Fig. 6:**
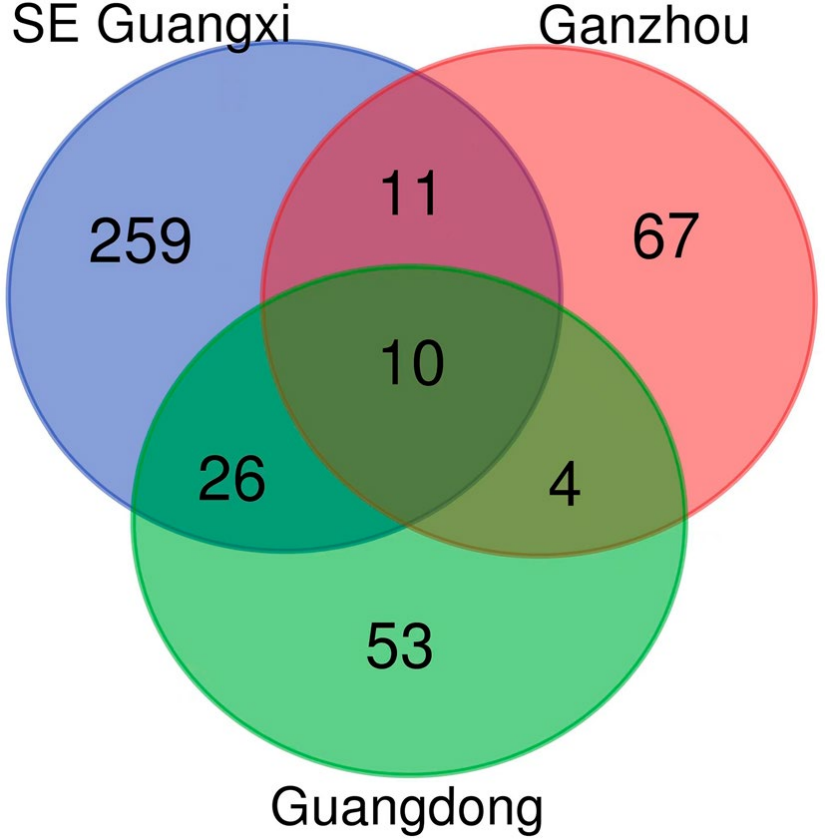
The comparative analysis of the overlap of medicinal plants in different Hakka areas shows the shared number of species within the groups

This study recorded 259 unique medicinal plant species, surpassing the records for Ganzhou (67 unique species) and Guangdong (53 unique species). Across the three sites, only 10 species were common, namely *Paederia foetida* L., *Liquidambar formosana* Hance, *Physalis angulata* L., *Ficus simplicissima* Lour., *Lophatherum gracile* Brongn., *Smilax glabra* Roxb., *Verbena officinalis* L., *Agrimonia pilosa* Ledeb., *Vitex negundo* and *Boehmeria nivea* (L.) Gaudich. Southeastern Guangxi and Guangdong share a higher number of species (36 unique species, Jaccard index [JI] = 0.1) compared to Southeastern Guangxi and Ganzhou (21 unique species, JI = 0.06) (Fig. 6).

## Discussions

### Traditional market medicine culture in the Hakka region of southeastern Guangxi

The Hakka people follow traditional Chinese medicine, adapt to local conditions, and use local materials due to their proximity to the remote area and local resources. With the efforts of generations of Hakka ancestors, they gradually explored the customs of disease prevention and treatment suitable for the local environment, forming a Hakka medical culture with strong regional characteristics [30]. Traditional markets are the showcase of tradition and culture [31].

Our study demonstrates that the use of traditional medicinal plants and selling culture in Hakka not only benefits the community’s health system but also serves as a source of income for the local people. However, the dependency on selling such plants is occasional (mostly in dragon festivals) for most people, with only a few selling medicinal plants throughout the year. The plants were sold in traditional ways such as in bundles or weight, and people were earning good sums of amounts through the sale of these medicinal herbs. Hence, medicinal plant collection and selling has been an income-generating option for people with low incomes and minorities [32]. They have good economic value, indicating their role in rural livelihood and income generation is important [33]. Most of the occasional vendors were farmers, which is positive for the sustainability of the local farmers continuing their occupation and passing the tradition to their future generations. However, it is worrisome that most vendors were over 60, which might cause problems in the vertical transfer of traditional knowledge systems. The vertical transfer of traditional knowledge has shaped the knowledge plants used in China and the world [34]. Hence, the younger generations should be promoting medicinal plant use and selling activities. The vertical knowledge of these traditional plants should be continued through enhancing interactions with the elder and young generations to continue important traditions and their knowledge [35]. These important practices and knowledge should be promoted and continued. They are important for promoting the Hakka culture and their expertise through vertical and horizontal knowledge transfer.

### Medicinal plants recorded

We recorded more medicinal plants than previous studies in Hakka communities [5, 28]. This is related to the number of sites included in this study and the focus of the studies on the traditional market. More records of medicinal plants might also be related to cultural adaptation acculturation [36]. The Hakka communities share living areas with the Cantonese. In other southeastern Guangxi regions not far from Bobai County and Luchuan County, such as Fangchenggang City and Guigang City, a significant portion comprises ethnic minorities, including the Yao and Zhuang peoples. The Hakka community has cultivated a rich Hakka traditional Chinese medicinal culture through sustained interaction with these diverse local groups. Further, the area's environment and remoteness have also supported a rich knowledge of medicinal plants used in the area. The medicinal plants reported by our studies are similar and more comprehensive than the previous reports on medicinal plants [37], food medicine such as soups [6, 38], and functional food [28]. These plants have a scope of promoting medicinal well-being, diet support, and food security [39].

Herbs were primarily used as medicine, followed by shrubs, trees, climbers, and parasites. The diverse number of species recorded in different categories has implications for preparing new drugs and supporting the treatment of old and new diseases such as the coronavirus [40]. Among the parts used, leaves use is a sustainable approach; however, using all the plant's aerial parts, whole parts, and roots is worrisome, particularly in trees. Using roots or whole plants poses a conservation threat to such plants [41]. The diverse plants recorded by our study have expanded the depth of traditional medicinal plants in the Hakka community. Hence, future research can focus on the sustainable use and management of such species.

Some plants recorded by this study are both medicine and food medicine. Studies have shown that medicinal plants with relatively high RI values have fewer side effects and are easier to obtain [27]. Local people are very familiar with plants with high RI value and their use in treating various diseases. For example, *Cordyline fruticosa* is used by Hakka people to treat injuries, digestive diseases, and hemorrhoids. It has multiple effects, including blood tonic, hemostasis, and diarrhea. It shows medicinal plants play a vital role in traditional Chinese medicine in the Hakka community in southeastern Guangxi and have potential development value.

### Patterns of medicinal plant use across disease categories

About 41% of the plants recorded were used in the treatment of physical trauma, including insect bites, snake bites, and other injuries of the bodies, which are the most common and easily known diseases ailment. Hence, people might have developed a more resilient system for easily detected diseases, similar to other studies [42]. Likewise, more than 30% of the medicinal plants recorded were for digestive disease ailments and local diseases. Few medicinal plants were reported for diseases like diabetes, anorexia, and cancer, as these diseases are not local and easily known diseases by local methods. Local communities have gone through a series of hits, trials, and successes for common diseases. However, hits and trials could not go through the long term for new forms of diseases and uncommon diseases. Hence, people might not have developed more medicinal alternatives for such diseases and ailments, and while using medicinal plants for complicated and new diseases, people should take more caution [40]. It is positive to record that nature and communities have diverse medicinal plants for treating different diseases and ailments, but precautions must be taken while using such plants. Consultation with an herbalist or traditional healer before using them is recommended.

### Comparison of traditional knowledge of medicinal plants in different Hakka regions

Our study reveals considerable variation in medicinal plant use among different Hakka communities. Specifically, the similarity index between the medicinal plant species recorded in the traditional markets of southeastern Guangxi and those documented in Hakka communities in Guangdong by Au et al. [5] is only 0.10, indicating a very low overlap in species composition [5].

The utilization of medicinal plants in a given region is not solely determined by local plant diversity but is also shaped by cultural, social, and economic contexts [43, 44]. Guangxi is a multi-ethnic province, home to 11 recognized minority groups, including the Zhuang and Yao, and to the Han majority. Through long-term cultural interaction and adaptation, the Hakka people who migrated to Guangxi have developed a unique system of traditional medicinal knowledge that reflects their heritage and influences from surrounding ethnic communities [45].

This intercultural exchange is evident in the widespread use of medicinal species traditionally associated with the Zhuang people, such as *Nanhaia speciosa*, *Ficus simplicissima*, and *Zanthoxylum nitidum*, also frequently found in Hakka markets. Similarly, local Hakka populations have partially adopted classification systems from Yao ethnomedicine. For example, *Kadsura coccinea* and *Kadsura long pedunculate* are, respectively, referred to as “da zuan” and “xiao zuan” by both Yao and Hakka communities. These species are used to dispel wind and dampness, promote blood circulation, alleviate pain, and stop bleeding—mainly in treating traumatic injuries and rheumatism. The influence of Yao ethnomedicine is further reflected in the extensive use of wind-dispelling herbs, which are common to both ethnic groups. Shared species include *Vitex negundo*, *Liquidambar formosana*, *Blumea balsamifera*, *Inula cappa*, and *Sarcandra glabra*.

Notably, our study differs from previous surveys by covering a wider range of market sites and incorporating longer-term ethnobotanical fieldwork, allowing for the documentation of more underreported and distinctive species. Environmental variation among sites likely contributes to differences in local plant availability and diversity [46], shaping plant use knowledge. Additionally, local socioeconomic conditions may influence the retention, transmission, and adaptation of ethnomedicinal practices [47].

### Challenges and opportunities of sustainability of medicinal plants

The medicinal plants recorded have primarily three benefits in terms of local context, which include support in rural revitalization through income generation, conservation of traditional knowledge, and conservation of plants. The Hakka communities of southeastern Guangxi have rich knowledge of medicinal plants. The systematic utilization of these plants supports rural revitalization through the trade of these plants [48]. The traditional market significantly supports conserving the traditional knowledge system, which will further foster the horizontal transfer of this knowledge to the community, which is important for conserving the traditional heritage. The medicinal plants useful for medicine will be planted or cultivated in home gardens, which will benefit and support the conservation of such plants [49].

The area faces three significant challenges: habitat degradation, obstruction of succession, and acculturation caused by the Yulin Chinese Medicine Port. The habitat of the many important plant species is degraded due to forest clearance and *Eucalyptus* plantations. Some medicinal plant resources that were more common in the past are now scarce. Of the 305 species recorded, only 104 species (34.09%) were listed under the global IUCN red list of threatened species, of which two were nearly threatened (NT) and one was vulnerable (VU). The nearly threatened species (*Eucalyptus robusta* and *Platycladus orientalis*) and vulnerable species (*Lithocarpus pachylepis*) should be used carefully to ensure their stable population. These species should be integrated into agroforestry activities, which could promote and conserve them. With the rapid decline of medicinal plant resources, the traditional knowledge related to them is gradually disappearing [50]. For example, some medicinal plants, *Capparis versicolor Dendrobium sp.*, *Tinospora sagittata, Cannabis sativa,* and *Melodinus cochinchinensis* were corded in the past as a treatment for various diseases [51]. Still, they were rarely mentioned by the respondents in this survey.

Moreover, with the rise of modern medicine and changes in lifestyle, people are losing faith in traditional Chinese medicine (TCM). This has led to the marginalization of rural doctors. Compounded by the family-centric “knowledge transmission within the family and not beyond” cultural practices, traditional medicinal knowledge faces vertical and horizontal transfer difficulties.

The Yulin Chinese Medicine Port is one of China’s largest markets for traditional medicinal plants[52]. The port provides convenience for the circulation of medicinal herbs, helps spread TCM knowledge, and introduces potential adverse effects. On the one hand, commercializing large-scale medicinal materials encourages the homogenization of medicinal plant products. This trend poses a risk to the traditional knowledge of medicinal plant diversity, especially as market demand grows for certain high-output, standardized medicinal materials. Suppliers and traders often focus on high-demand, mass-produced herbs, which neglect local, specific medicinal plants. As a result, some locally used plants and the traditional knowledge associated with them could be overshadowed or replaced by mainstream market products. On the other hand, the market operation requires an ample supply of medicinal plants, which leads to the large-scale and low-price resale of many wild plants to the Yulin Chinese Medicine Port Market. This has resulted in the gradual decrease of some common medicinal plants in the local area, which is not conducive to the sustainable utilization of local medicinal plant resources.

Thus, while the Yulin Chinese Medicine Port fosters the circulation of medicinal herbs, its tendency toward standardization may undermine the diversity of traditional medicinal plants and their knowledge. The sustainable use and conservation of local medicinal plants play a vital role in preserving and transmitting ethnobotanical knowledge [53]. The sustainable use of the plants can be achieved by strengthening cooperation among the government, research institutions, and enterprises, using modern scientific methods to ensure the continued application of traditional medicinal plants in modern society while preserving their cultural and ecological significance.

### Limitations of the study

This study is primarily based on the interview of medicinal plants knowledge with the traditional Hakka shopkeepers in the traditional medicine market in southeastern Guangxi. This work has only covered the documentation of medicinal plants in the traditional markets, assessed the ethnobotanical value of each plant in the market, and compared it with similar studies in another region. Future studies can focus on taking the data from sellers and non-sellers, conducting economic analysis of each plant in the area, the factors influencing the plants used and sold, and the pharmacological relevance of the plants used in medicine.

## Conclusion

The traditional Hakka medicine market in southeastern Guangxi is rich in medicinal plant resources with both therapeutic and nutritional value. These markets play a vital role in preserving local knowledge and supporting community livelihoods. However, the frequent harvesting and sale of herbs—particularly stems, roots, and whole plants—raise conservation concerns. Commonly used species tend to show higher relative importance (RI) values than rare ones. Challenges such as land use change, weakened knowledge transmission, the influence of modern medicine, and declining local interest threaten the sustainability of both the markets and the associated traditional knowledge. Nevertheless, the proximity to regional hubs like the Yulin Chinese Medicine Port presents opportunities to expand trade and enhance economic value. We recommend integrating native species into cultivation practices, strengthening knowledge transmission, and promoting product development and public awareness to ensure the long-term sustainability of these ethnobotanical resources.

## Data Availability

No datasets were generated or analysed during the current study.
